# Consistent time allocation fraction to vegetation green-up versus senescence across northern ecosystems despite recent climate change

**DOI:** 10.1126/sciadv.adn2487

**Published:** 2024-06-07

**Authors:** Fandong Meng, Andrew J. Felton, Jiafu Mao, Nan Cong, William K. Smith, Christian Körner, Zhongmin Hu, Songbai Hong, Jonathan Knott, Yanzi Yan, Bixi Guo, Ying Deng, Stephen Leisz, Tsechoe Dorji, Shiping Wang, Anping Chen

**Affiliations:** ^1^ State Key Laboratory of Tibetan Plateau Earth System, Resources and Environment (TPESRE), Institute of Tibetan Plateau Research, Chinese Academy of Sciences, Beijing 100101, China.; ^2^ Department of Land Resources and Environmental Sciences, Montana State University, Bozeman, MT 59717, USA.; ^3^ Environmental Sciences Division and Climate Change Science Institute, Oak Ridge National Laboratory, Oak Ridge, TN 37831, USA.; ^4^ Key Laboratory of Ecosystem Network Observation and Modeling, Institute of Geographic Sciences and Natural Resources Research, Chinese Academy of Sciences, Beijing 100101, China.; ^5^ School of Natural Resources and the Environment, University of Arizona, Tucson, AZ 85719, USA.; ^6^ Department of Environmental Sciences, Botany, University of Basel, Basel, Switzerland.; ^7^ Key Laboratory of Agro-Forestry Environmental Processes and Ecological Regulation of Hainan Province, Hainan University, Haikou, Hainan 570228, China.; ^8^ School of Urban Planning and Design, Shenzhen Graduate School, Peking University, Shenzhen 518055, China.; ^9^ USDA Forest Service, Northern Research Station, Forest Inventory and Analysis Program, St. Paul, MN 55108, USA.; ^10^ Department of Physical Geography and Ecosystem Science, Lund University, Lund, Sweden.; ^11^ State Key Laboratory of Vegetation and Environmental Change, Institute of Botany, Chinese Academy of Sciences, No. 20 Nanxincun, Xiangshan, Beijing 100093, China.; ^12^ Department of Anthropology and Geography, Colorado State University, Fort Collins, CO 80523, USA.; ^13^ College of Arts and Sciences, Vin University, Gia Lam, Hanoi, Vietnam.; ^14^ Department of Biology and Graduate Degree Program in Ecology, Colorado State University, Fort Collins, CO 80523, USA.

## Abstract

Extended growing season lengths under climatic warming suggest increased time for plant growth. However, research has focused on climatic impacts to the timing or duration of distinct phenological events. Comparatively little is known about impacts to the relative time allocation to distinct phenological events, for example, the proportion of time dedicated to leaf growth versus senescence. We use multiple satellite and ground-based observations to show that, despite recent climate change during 2001 to 2020, the ratio of time allocated to vegetation green-up over senescence has remained stable [1.27 (± 0.92)] across more than 83% of northern ecosystems. This stability is independent of changes in growing season lengths and is caused by widespread positive relationships among vegetation phenological events; longer vegetation green-up results in longer vegetation senescence. These empirical observations were also partly reproduced by 13 dynamic global vegetation models. Our work demonstrates an intrinsic biotic control to vegetation phenology that could explain the timing of vegetation senescence under climate change.

## Teaser

Plants maintain stable time allocation amid climate change to balance durations of growth and senescence.

## INTRODUCTION

The allocation of resources in plants is a fundamental aspect of their growth, exerting a strong influence on their life cycle and the structure and function of terrestrial ecosystems (*1*–*3*). Resource allocation in plants involves the distribution of carbon, water, and nutrients, among different organs, to perform different functions (*4*). The allocation of these physical resources has received abundant attention in the literature [e.g., (*5*–*7*)]. On the other hand, the allocation of time—perhaps the most limiting resource of all—has received unexpectedly limited attention, although individual events (e.g., leaf out) related to the timing of plant growth have been extensively studied (*8*–*11*). Time as a resource is perhaps most pertinent to plants experiencing annual phenological cycles, often defined by growing seasons. A growing season’s duration can further be divided into different plant growth stages (*12*); each stage is allocated to a specific portion of the overall time period, which we will refer to as “allocation of periods of time” (hereafter time allocation). For instance, at the species level in two divergent alpine grasslands, warming prolongs the reproductive period more than the vegetative phase (*13*) but does not extend the leaf growth period (*14*). These results imply an adjustment of time allocation of plants to adapt to environmental change through enhanced time allocation to reproduction. However, the time allocation among different phenological durations has been rarely tested in larger regions.

The impact of climate change on plant phenology has been extensively investigated as a notable biological response to, and an indicator of, global environmental change (*15*–*18*). For example, the individual timing of phenological events, like spring green-up, has contributed greatly to our comprehension of ecosystem responses to climate change (*9*, *19*, *20*). However, a more complete picture of climate change impacts on phenology would require understanding the response of a series of phenological events. For instance, reproductive time allocation encompasses the durations of flower bud development, flowering, and seed production during the growing season. These diverse phenological events exhibit varying responses to climate change (*21*), and the life cycle of each plant organ is influenced by relative changes in their initiation and cessation of growth. Furthermore, in addition to climate change, plants’ self-regulation also controls phenological variation as alterations in preceding phenological events affect subsequent ones (*13*, *22*). A comprehensive examination of the roles played by these drivers in vegetation time allocation is crucial for understanding their time allocation patterns under climate change (*3*, *23*, *24*).

**Fig. 1.**
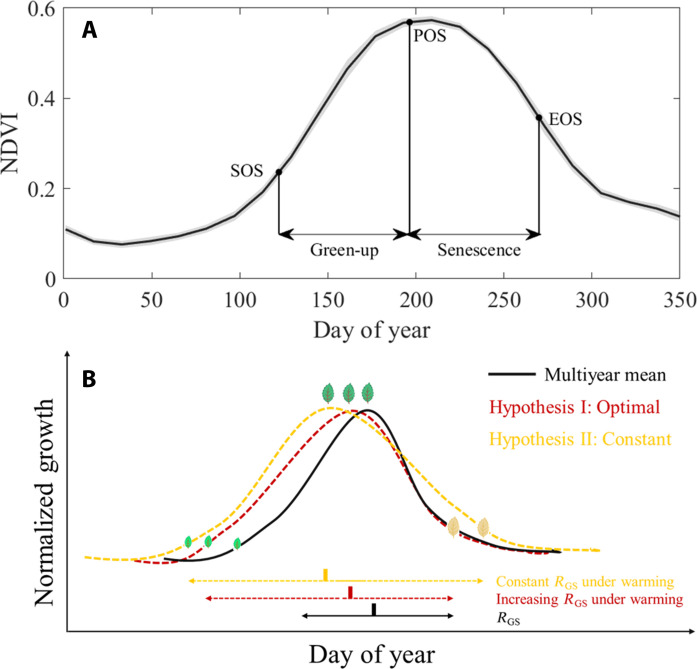
Conceptual diagram of time allocation between VGU and VSS in northern ecosystems (>30°N). (**A**) The black lines represent the multiyear average NDVI, and dots represent timing of phenological events, including the SOS, POS, and EOS across northern ecosystems. The gray areas indicate the standard deviations (SDs) of interannual fluctuations in NDVI. The duration of VGU is defined as the time span from SOS to POS, while the duration of VSS is defined as the time span from POS to EOS. Then, *R*_GS_ is the ratio of durations of VGU to VSS. (**B**) This figure depicts two examples of potential time allocation strategies under warming conditions; other potential changes in phenology under warming and cooling are not shown in the figure. The black and warm colored curves represent the normalized growth trends during normal and warming years, respectively. Note that this study does not consider the magnitude of growth, so all curves have the same peak values in (B). Hypothesis I, represented by the red dashed line, suggests optimal time allocation. Hypothesis II, depicted by the yellow dashed line, proposes constant time allocation. The double-headed arrows indicate changes in the durations of VGU and VSS.

Here, we examine trends in the time allocation of different vegetation phenological durations in northern ecosystems, which covers divergent vegetation types including trees, shrubs, and grasslands with distinct annual phenological cycles. To this end, we used satellite remote sensing datasets, including normalized difference vegetation index (NDVI) data derived from the moderate resolution imaging spectroradiometer (MODIS) and the contiguous solar-induced chlorophyll fluorescence (CSIF) data, covering the period from 2001 to 2020. Here, CSIF is the fusion of Orbiting Carbon Observatory-2 (OCO-2) solar-induced chlorophyll fluorescence (SIF) data and MODIS reflectance data. Traditional ground-based observations of phenology focus on the specific timing of different events [e.g., flowering; (*15*)], whereas satellite-based observations offer the advantage of monitoring changes in greenness as well as photosynthetic activities throughout the growing season on a large scale (*15*, *17*, *25*). We used the entire canopy to serve as a “big leaf” representing the three phenological timings including the start of the growing season (SOS), peak of the growing season (POS), and end of the growing season (EOS) that are identified (see Materials and Methods). From these, we calculated the time allocation to vegetation green-up (VGU), which represents the period from SOS to POS, and vegetation senescence (VSS), which represents the period from POS to EOS (Fig. 1A). We then quantified vegetation phenology time allocation at the ecosystem level as the ratio of VGU to VSS, denoted as *R*_GS_.

We tested two contrasting hypotheses regarding vegetation phenology time allocation. The first hypothesis, which we termed the optimal time allocation hypothesis, suggests that vegetation time allocation changes in response to variations in environmental conditions over time. A longer VGU period, which may indicate lower photosynthesis rates and potential higher stress resistance (*26*–*28*), may be more advantageous in regions with frequent climate fluctuations. However, favorable environments can accelerate plant growth because early growth promotes carbon assimilation (*29*, *30*) and more carbon could be allocated to leaves (*31*) to advance the peak growth (*8*, *11*). This would result in a shorter VGU period (hypothesis I in Fig. 1B). Therefore, according to this hypothesis, we expect a temporal trend in time allocation as a response to climate warming, implying that this allocation is not biologically regulated but rather plastic.

An alternative hypothesis, which we termed the constant time allocation hypothesis, suggests a relatively stable time allocation due to a carryover effect among the timing and duration of other phenological events (*32*), implying that this allocation is intrinsically biologically regulated. For example, an earlier SOS could lead to an earlier POS. Also, this earlier POS and more carbon uptake could further lead to an advance of EOS mainly through the mechanism of sink limitation as more assimilated carbon may exceed the need for plant growth (*10*). These cascading effects could result in an earlier EOS, hence maintaining a consistent ratio between the durations of VGU and VSS (hypothesis II in Fig. 1B). We also validated this remotely sensed–derived pattern on vegetation phenology time allocation using other empirical estimates of vegetation phenology from PhenoCam and FLUXNET eddy covariance tower sites. We further explored how temporal trends in time allocation may be influenced by environmental and physiological factors based on observations and 13 dynamic global vegetation models (DGVMs) participating in the project “Trends in the land carbon cycle” (TRENDY). Last, we investigated if time allocation could explain the timing of VSS.

## RESULTS

### Temporal trend in vegetation phenology time allocation ratio

**Fig. 2.**
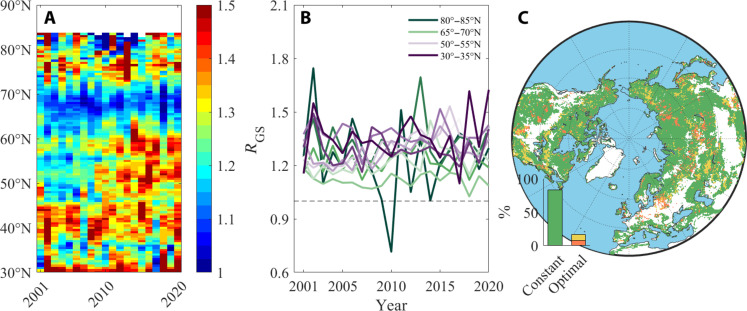
Temporal trend in time allocation derived from remotely sensed data. (**A**) The Hovmöller diagram displays the time series of time allocation between VGU and VSS across latitudes in northern ecosystems (>30°N). The duration of VGU is defined as the time span from SOS to POS, while the duration of VSS is defined as the time span from POS to EOS. (**B**) Temporal trend in vegetation time allocation based on NDVI. Only in the 45° to 55°N latitude band that the temporal trends of vegetation time allocation had *P* values less than 0.05. The dashed line is *R*_GS_ = 1. (**C**) Different temporal trends of vegetation time allocation for each pixel. The constant and optimal time allocation strategies are represented by green and red/yellow colors, respectively. Green indicates nonsignificant trends, while red/yellow represents significant linear/nonlinear trends. The inserted histograms display the frequency distribution of each time allocation pattern. Further details about the two time allocation hypotheses are described in Introduction.

Across northern ecosystems (>30°N), 34.8% of the pixels experienced significant changes in the timing or duration of phenological events between 2001 and 2020 (*P* < 0.05; fig. S1). Growing season length increased across 62.3% (16.5% significant at the *P* < 0.05 level) of the area and was due to extended durations in both VGU and VSS (fig. S1). The time allocation coefficient, or *R*_GS_, was approximately 1.27 (± 0.92) across the entire northern ecosystems (fig. S2). This indicates that these ecosystems, on average, experience a longer duration of VGU compared to VSS. With the exception of the 45° to 55°N latitude band, the temporal trend of the time allocation coefficient displayed an insignificant trend and thus a relatively stable ratio (*P* > 0.05), regardless of whether the length of the growing season was prolonged or not (Fig. 2, A and B).

We found strong evidence for the constant time allocation hypothesis (Fig. 1B). In 83.3% of the pixels, there were no significant temporal trends in *R*_GS_ (*P* > 0.05), regardless of changes in the length of the growing season (Fig. 2C and fig. S2). By contrast, we found support for the optimal time allocation hypothesis (Fig. 1B) in only 7.7% of the pixels, where there was a significant linear trend of *R*_GS_ over time (*P* < 0.05), with the small sample-corrected Akaike information criterion (AICc) of the linear model being lower than that of the piecewise linear model (a lower AICc, a better model; Fig. 2C and fig. S2). In addition, in 8.9% of the pixels, there was a significant piecewise linear trend of *R*_GS_ over time (*P* < 0.05), with a lower AICc than that of the linear model (Fig. 2C and fig. S3), also supporting the optimal time allocation hypothesis. Furthermore, we integrated the CSIF index, which serves as a proxy for vegetation photosynthesis, into our analysis. The variation in CSIF is closely related to that in NDVI (figs. S4 and S5), while it also provides a high temporal resolution with distinct seasonality (*33*). We found similar results supporting the constant time allocation hypothesis when analyzing the CSIF index from 2001 to 2020 (fig. S6). Specifically, results were consistent with the constant time allocation hypothesis in 83.3% of the study area. We also used a lower statistical threshold (*P* < 0.01) and a longer time-span dataset (34 years of the Global Inventory Modeling and Mapping Studies (GIMMS) NDVI data), which returned similar results (fig. S7). Overall, these results show that changes in growing season lengths across the Northern Hemisphere in the 21st century have had little impact on the time allocation between durations in VGU and VSS and further suggest that this time allocation is biologically controlled.

We next applied multiple methods to evaluate the robustness of our finding of widespread constant *R*_GS_ (hypothesis II). First, we applied various phenological extraction algorithms (fig. S8) as well as a land surface phenology product (MCD12Q2 v6.1) generated with a different algorithm (fig. S9). This analysis further supported the near ubiquity of the constant time allocation hypothesis. Second, we explored the influence of three environmental factors (temperature, moisture, and radiation) and growing season length on time allocation. Despite variations in these factors over the study period, the results demonstrated that a constant time allocation persisted in most regions, with percentages ranging from 68.5 to 86.3% (figs. S10 and S11). This suggests that the constant proportion may not arise from mutual compensation of divergent effects among different environmental and biotic factors on *R*_GS_. Third, in addition to nonlinear analysis, we assessed the stationarity of the time series by comparing mean values and standard deviations (SDs) between two periods (2001 to 2010 versus 2011 to 2020). No significant differences (*P* > 0.05) were found in mean values (93%) and SDs (70%) between these periods in the northern ecosystem (fig. S12). This further supports the hypothesis of constant time allocation. Fourth, we evaluated the anomaly values of time allocation by subtracting their multiyear averages to account for potential vegetation age-related effects. Even with this approach, the constant time allocation hypothesis remained supported (fig. S13). Last, considering the observed steady period in ground leaf life span besides leaf formation and leaf senescence, we also classified the vegetation life span into three periods (see Materials and Methods); the stable time allocation still persisted in 85.8% of the study area (fig. S14).

**Fig. 3.**
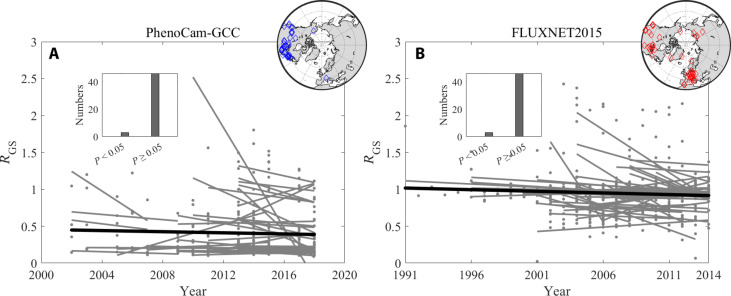
Temporal trend in vegetation phenology time allocation at 50 PhenoCam and 49 FLUXNET2015 sites. The inset circles indicate the geographical locations of 50 PhenoCam sites (**A**) and the 49 FLUXNET2015 flux tower sites (**B**). The black line represents an overall fitted trendline, and the other gray lines represent fitted trendlines at the individual sites. The inset bar charts are the site numbers with *P* values of linear regressions of *R*_GS_ larger or less than 0.05.

We then used data from the PhenoCam greenness phenology network (here 50 sites) and the FLUXNET2015 photosynthesis phenology dataset (49 sites) to calculate the time allocation ratio, focusing mainly on North America and Europe (table S1 and Fig. 3). Because of the relatively short durations of data, we used a linear model to examine the temporal trend of time allocation. Our findings revealed that only 6.0% (3 out of 50 sites) and 6.1% (3 out of 49 sites) of the PhenoCam and FLUXNET2015 sites exhibited a significantly negative linear trend, respectively (*P* < 0.05). On the other hand, most sites displayed rather constant *R*_GS_ (table S1 and Fig. 3). We further explored the dynamics of and potential changes to the growth curve shape, which also exhibited little changes over time (fig. S15). Collectively, our findings provide robust evidence for the constancy of the time allocation pattern between durations of VGU and VSS as well as the growth pattern from 2001 to 2020, despite variations in vegetation activity and directional changes to climate.

### Determinants of vegetation phenology time allocation changes

**Fig. 4.**
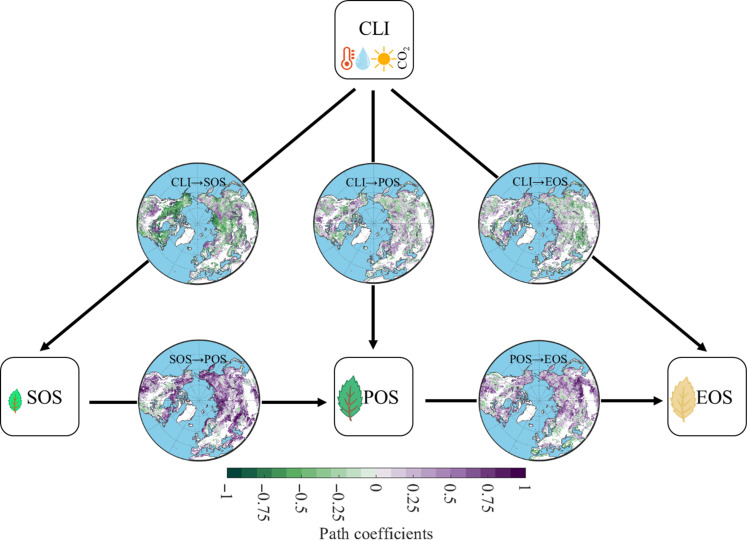
Effect of each factor on timings of three phenological events by PLS-PM analysis. CLI is a combined variable including annual mean temperature, precipitation, radiation, and CO_2_. The arrow (→) describes the causal relationship. For example, CLI→SOS is the effect of CLI on SOS; a negative path coefficient between CLI and SOS indicates that an increase in CLI advances SOS, and vice versa. SOS is the start of the growing season, POS is the peak of the growing season, and EOS is the end of the growing season. The goodness of fit of the PLS-PM model is shown in figs. S16 and S17.

To understand how the three timings of phenological events (SOS, POS, and EOS) dominate the time allocation ratio between durations of VGU and VSS (*R*_GS_), we used partial least squares path modeling (PLS-PM) combined with climatic factors and three timings of phenological events (see Materials and Methods for details). Our findings revealed strong positive effects of the earlier timing of phenological events on the latter one. Specifically, SOS had a positive effect on POS (in 84% of the studied area and with a mean value of 0.36 ± 0.34), and POS had a positive effect on EOS (in 77% of the studied area and with a mean value of 0.24 ± 0.33; Fig. 4 and fig. S16 and 17). These intrinsic interactions among timings of phenological events were found to have a greater impact than climatic factors in most regions of the Northern Hemisphere (Fig. 4 and fig. S18). Thus, the preceding phenological events exhibited a stronger influence on subsequent events than climatic factors. Partial correlation analysis yielded similar results, albeit with a lower degree of impact compared to PLS-PM, which could be attributed to its inability to isolate indirect effects (fig. S19).

**Fig. 5.**
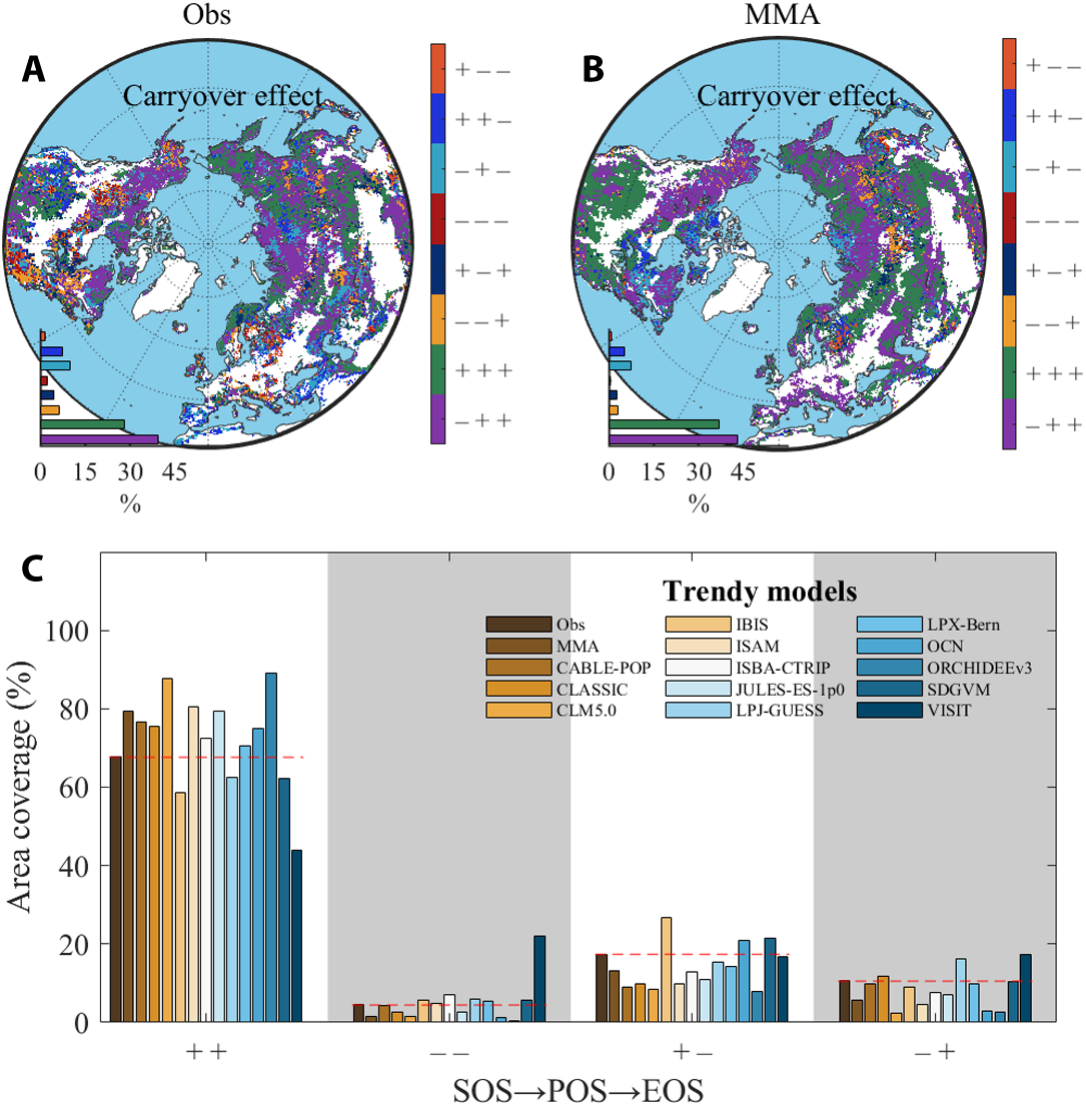
Spatial pattern of carryover effect of CLI→SOS→POS→EOS. The carryover effect is calculated by PLS-PM analysis based on satellite observations (Obs) (**A**) and multimodel average (MMA) gross primary productivity (GPP) of 13 TRENDY models (**B**). For example, “− + +” represents negative correlations between climate variables and SOS (CLI→SOS) and positive correlations between SOS and POS (SOS→POS) and POS and EOS (POS→EOS). (**C**) Area coverage of four patterns of SOS→POS→EOS. The red dashed lines are the observed area coverage of each carryover effect.

This vegetation phenology carryover effect, represented by the positive relationship of SOS→POS→EOS (+ +), contributed to a consistent pattern of vegetation phenology time allocation in support of the constant time allocation hypothesis across most northern ecosystems (68%; Fig. 5A) while the other three patterns only accounting for 32% of the area (i.e., + −, − +, and − −).

Next, we tested the ability of 13 TRENDY v9 models in capturing the carryover effect. Overall, although the area proportion with modeled carryover effect ranges from 44 to 89% across different models (fig. S20), the multimodel average of the 13 models well replicated the spatial pattern of the observations (with a coverage of 79% for models versus 68% for observations; Fig. 5, A and B). Regions with overestimated carryover effect (+ +) are mainly found in eastern North America (Fig. 5). These results collectively indicate that the time allocation of vegetation phenology to different growth stages appears intrinsically biologically controlled across much of the Northern Hemisphere.

### Predicting EOS based on the constant time allocation hypothesis

**Fig. 6.**
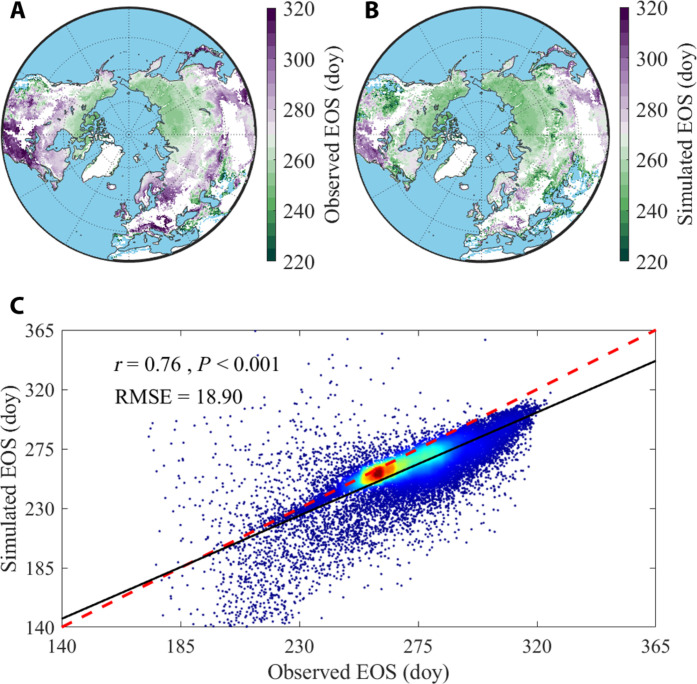
Spatial pattern of EOS across northern ecosystems. Spatial pattern of observed EOS (**A**) and simulated EOS (**B**). doy, day of year. (**C**) Simple linear relationship between observed and simulated EOS across northern ecosystems. The color gradients from blue to red represent increased density of points. RMSE is used to estimate the performance of model. The significant level is at the 0.05 level.

The constant vegetation phenology time allocation hypothesis represents the quantitative relationships among timings of the three phenological events, irrespective of climate change. To further examine this hypothesis, we conducted simulations of timing of autumn phenology using the relationships derived from constant vegetation phenology time allocation and timing of spring/summer phenology (see Materials and Methods for details). The results demonstrated a similar spatial pattern between the observed and simulated EOS (Fig. 6, A and B). The simple process-based model accounted for 58% of the observed EOS variability (*r* = 0.76, *P* < 0.001), with an accuracy of 18.90 days [root mean square error (RMSE); Fig. 6C]. Similar results were obtained using median time allocation ratios (fig. S21). In addition, they also had a similar temporal trend with an area coverage of 75% (31% at the significant level of *P* < 0.05; fig. S22). Although the model tended to underestimate EOS in low latitudes, particularly in European regions, the observed and simulated EOS closely matched in high latitudes (fig. S23). Overall, the prediction of EOS based on the constant vegetation phenology time allocation hypothesis effectively captured the spatial pattern of observed EOS in ecosystems across the Northern Hemisphere.

## DISCUSSION

Our most important finding is that of evidence for an intrinsic biological control in how time is allocated to different durations of phenological stages of vegetation growth and senescence across highly seasonal northern ecosystems. Despite extended growing season lengths and the longer leaf life span with climatic warming (*12*, *34*), we observed that there has been no evident change in the relative allocation of time to either duration of VGU or VSS in the past two decades (*14*). This observation supports our hypothesis of a constant time allocation pattern (Fig. 1B) and was consistent across multiple data sources including NDVI (Fig. 2), SIF observations (fig. S6), and a comprehensive analysis of phenology from PhenoCam and FLUXNET2015 (table S1 and Fig. 3). This constant time allocation pattern can be attributed to several underlying processes that are themselves future hypotheses to test. Spring phenology, as the earliest phenological event in the growing season, is particularly sensitive to climate changes (*35*). Our analysis revealed a substantial advancement of spring phenology in most regions of the Northern Hemisphere due to climate change, primarily driven by warming, which alleviates low-temperature limitation (*12*, *36*). However, in certain regions characterized by water limitation on vegetation growth, such as the Great Plains of North America, Central Asia, and the Inner Mongolia Plateau, spring phenology has shown a delaying trend and could be further delayed by exacerbated water scarcity under future warming (*37*, *38*). While the response of the latter two timings of phenological events (POS and EOS) to climate change varied across regions (fig. S1), their timings were predominantly influenced by the carryover effect from the preceding phenological event (Figs. 4 and 5). This suggests that the interactions and dependencies between successive phenological events play a crucial role in shaping the constant time allocation pattern.

The observed carryover effect in the SOS→POS→EOS relationship may be attributed to two underlying mechanisms. First, there is an endogenous carryover effect that is often mediated by biological or physiological processes (*39*, *40*). The advancement of spring phenology stimulates plants to accumulate more carbohydrates, potentially through hormonal control (*32*) or freezing tolerance (*40*). This increased carbohydrate availability promotes the advancement of POS (*8*, *11*), potentially through enhanced leaf carbon allocation (*31*). Conversely, the positive relationship between the POS and EOS can be explained by a source-sink effect. When the production of carbohydrates (source) exceeds the capacity of various plant organs to use them (sink), negative feedback mechanisms are triggered, leading to accelerated EOS. This phenomenon has been widely observed and validated in herbaceous plants and trees (*10*, *41*–*43*). The endogenous carryover effect may also be influenced by genetic programming (*44*), such as allometric relationships that may determine vegetation phenology time allocation. In addition, the extended recovery time for carbohydrates or nutrients may be required for higher productivity associated with a longer VGU period (*45*). The existence of this carryover effect has been demonstrated in many studies (*22*, *32*, *46*–*48*). However, most of these studies focused on individual phenological events rather than considering the interactions among multiple phenological events. Overall, it is the combination of these carryover effects in the SOS→POS→EOS relationship that contributes to the establishment of the fixed time allocation pattern.

In addition, there may also be an exogenous carryover effect that is primarily controlled by environmental or abiotic factors (*39*). The early and intensified vegetation activity in spring can lead to summer drought conditions (*37*, *49*), resulting in an advancement of the POS (*11*). Similarly, the increased water and nutrient consumption, including nitrogen, by the POS can also accelerate the arrival of the EOS (*50*–*53*). However, our PLS-PM analysis in this study differentiated the coupling effects of previous timings of phenological events and current climate factors and found that the influence of climate factors on current timings of phenological events was relatively weaker. Therefore, the dominant role in the timing of phenological events was attributed to the endogenous carryover effect rather than the exogenous one. In addition to the observed constant time allocation pattern, we also identified regions with an optimal time allocation pattern, mainly concentrated in the middle latitudes (45° to 55°N) with a coverage of 16.6% (Fig. 2). These regions are predominantly characterized by deciduous broadleaf forests and mixed forests (54.2%) (fig. S24). Notably, in these regions, there was a tendency to allocate more time to VGU than to VSS over time (figs. S2 and S25). This allocation pattern may enhance stress resistance in response to frequent extreme heat and rainfall events that occur in these regions (*54*, *55*), aligning with our first hypothesis.

Our study presents an analytic framework highlighting the concept of constant time allocation among timings or durations of phenological events, emphasizing the need to consider interconnected plant growth processes rather than isolated phenological events (*13*, *23*). Additionally, we tested the ability of 13 TRENDY models to capture the observed widespread carryover effect (Fig. 5 and fig. S20). The reason why the model cannot accurately reproduce and overestimate the observation results in the eastern United States (+ +) is unknown. One possible reason is that the models do not accurately describe the impact of environmental factors on SOS, which is also previously demonstrated by 18 terrestrial biosphere models (*29*). Because most phenological modules use a simple temperature accumulation model (e.g., growing degree day), it may affect prediction effectiveness due to other combined factors and frequent extreme climates (*51*, *53*, *56*), for example, frequent flash droughts in northeastern North America (*55*).

Although the mechanism of SOS→POS→EOS (+ +) was not clearly parameterized in these models, it could still be found in vegetation growth time, particularly in Eurasia. This gives us confidence in quantifying and applying this mechanism explicitly. The constant time allocation pattern has the potential to address uncertainties in current phenological research. For instance, while spring phenology tends to advance with climate warming, changes in autumn phenology exhibit heterogeneity (*18*, *56*). The complexity of factors influencing autumn phenology, such as temperature, photoperiod, CO_2_, early phenological events, and carbohydrate accumulation, makes its prediction challenging (*10*, *32*, *51*, *57*–*59*). However, incorporating the constant time allocation pattern into phenology models is expected to enhance predictive capabilities. Our initial attempts to predict autumn phenology using this pattern show promising results (Fig. 6). However, we found that the EOS was underestimated in some regions especially the European region, which may be driven by other physiological mechanisms such as vegetation productivity (*10*). We further compared the relative importance between carryover effect and productivity (here represented by SIF as a proxy) on EOS. In most regions, the carryover mechanism outweighed the impact of productivity on EOS, while the European region was mainly dominated by productivity, aligning with prior experimental findings (fig. S26) (*10*). This pattern may enable plants to maintain optimal fitness in a changing environment by avoiding excessive resource investment in a single growth stage.

There are several limitations that should be considered when generalizing the findings of this study. First, while our results are supported by different phenological extraction algorithms and remotely sensed indices, the physiological significance of remotely sensed vegetation phenology is not fully understood. It remains challenging to quantitatively integrate remotely sensed findings with direct ground-based phenological observations (*1*, *60*). Alternatively, the abundance of different species with different phenological patterns has an influence on the signal of remote sensing, which also challenges the remotely sensed findings (*12*, *61*). There is thus an urgent need for ground-level data on qualitative and quantitative time allocation to complement our findings. In addition, satellite-based datasets may introduce uncertainties due to factors such as satellite drift, sensor aging, distinctions between different sensors, and atmospheric aerosol effects (*16*) as well as due to the spatial resolution of the sensor (*62*). For example, the datasets used here were at a temporal resolution of 16 days and a spatial resolution of 0.5°, which may introduce substantial uncertainties due to its relatively coarse resolutions that average over important information. For instance, in areas of ecological transition with mixed plant functional types, consistent leaf time allocation might be attributed not only to an intrinsic biological constraint but also to shifts in vegetation type across spatiotemporal grains due to divergent growth timings of plants. Therefore, it is essential for future research to investigate the phenomena and potential mechanisms highlighted in our work at a finer scale to eliminate the potential scale effect in ecological studies (*63*, *64*).

Second, our knowledge regarding time allocation in other plant organs is limited. For instance, data on reproductive phenology, which plays a crucial role in plant reproduction and food resources (e.g., seed production), are lacking (*65*). Therefore, understanding the time allocation among durations of reproductive phenological events (e.g., flowering and fruiting) as well as the allocation between durations of reproductive and vegetative phenology and even between durations of aboveground and belowground phenology, is essential for a comprehensive understanding of plant growth. Roots may also adopt a conservative pattern in response to warming, as shown in alpine grassland (*14*). However, further verification on a larger scale is still required. Last, it remains uncertain whether the current time allocation pattern will remain constant or undergo changes in response to continued warming and extreme events. The impact of ongoing environmental changes on the stability of the constant time allocation pattern requires further investigation. To investigate the influence of long-term climate and extreme weather on *R*_GS_, we used elevation as a proxy. Elevation change is associated with a well-established lapse rate of temperature change (~0.6°C per 100 m). Our analysis revealed that an evident increase in *R*_GS_ only occurred at elevations above 3000 m (fig. S27A). However, it is important to note that high-elevation areas (above 3000 m) account for only ~3% of the Northern Hemisphere (fig. S27B), mainly on the Tibetan Plateau in China (fig. S27C). This result provides further support for our first hypothesis, suggesting that harsher environmental conditions may lead to a greater time allocation to green-up. Future studies should aim to address these limitations and expand our understanding of plant growth dynamics in the face of changing environmental conditions.

Using satellite-observed timings and durations of vegetation phenology and corroborated with phenology from PhenoCam and FLUXNET2015, we have revealed that the constant time allocation pattern predominates in northern ecosystems. We have shown that, despite the varying responses of different timings of phenological events to climate change, there exists a consistent time allocation pattern between durations of VGU and VSS due to positive relationships among timings of phenological events, which is also partly supported from 13 DGVMs. However, extreme climate events (e.g., drought) may alter this constant time allocation, as shown by the comparison between the results from observations and DGVMs. This study presents a conceptual framework for understanding the biological mechanisms of phenology and quantifying the intrinsic connections among phenological events. Under the biological control of constant time allocation, the advance in early phenology due to future warming would offset the delay trend in autumn phenology caused by warming. This may limit its ability to fully benefit from future continued warming, potentially leading to increased carbon emissions through ecosystem respiration. Further studies focusing on ground observations of reproductive time allocation could provide valuable insights to complement this framework.

## MATERIALS AND METHODS

### Satellite-based vegetation phenology

The NDVI is a commonly used indicator of vegetation greenness, which allows for the study of vegetation phenology dynamics in northern ecosystems (>30°N) with a clear seasonal pattern. The region is mainly covered by deciduous forests, open shrublands, and grasslands. In this study, we used the MODIS NDVI data (specifically, the MOD13C1 version 6 product) obtained from the following source: https://lpdaac.usgs.gov/products/mod13c1v006/. This dataset provides a spatial resolution of 0.05° and a temporal resolution of 16 days, covering the period from 2000 to the present. SIF was also used in our study, which is closely related to vegetation photosynthesis, and was obtained from a contiguous SIF product derived using machine learning techniques (neural network) (*33*). This dataset has a temporal resolution of 4 days and a spatial resolution of 0.05°, covering the period from 2000 to the present. We acquired the data from the following source: https://doi.org/10.17605/OSF.IO/8XQY6. We also used the GIMMS NDVI dataset for its longer time span from 1982 to 2015 (*66*, *67*). This dataset provides a spatial resolution of 0.083° and a temporal resolution of fortnight (https://climatedataguide.ucar.edu/climate-data/ndvi-normalized-difference-vegetation-index-3rd-generation-nasagfsc-gimms).

To quantify the time allocation of vegetation phenology between the durations of VGU and VSS, we extracted timings of three key phenological events for each pixel-year combination throughout the growing season. These events include the SOS, the POS, and the EOS. Specifically, we defined the VGU as the duration between POS and SOS (SOP) and the VSS as the duration between EOS and POS (POE). The time allocation of vegetation phenology was then calculated as the ratio between SOP and POE, i.e., ratio = (POS-SOS)/(EOS-POS). In addition, the observed ground leaf formation always slows down above 80% of the maximum NDVI (*68*), then experiencing a stable or operation stage before senescing (*12*). To examine the potential effect of definition of these stages on the results, we used a 90% threshold of the maximum NDVI as the date of the end of VGU in the left of operation time [pre-POS (PPOS)] and as the date of the start of VSS in the right of operation time [after-POS (APOS)]. The time allocation of vegetation phenology was then calculated as ratio = (PPOS-SOS)/(EOS-APOS).

To ensure the accuracy and reliability of our phenological extraction, we used three commonly used algorithms: HANTS, POLYFIT, and DLOG, which have been previously validated (table S2) (*25*, *69*, *70*). These algorithms help mitigate uncertainties associated with fitting curves and improve the robustness of our results. The phenological extraction process consisted of two steps. First, we applied each algorithm to fit the original NDVI dataset at each pixel, generating a continuous time series of daily NDVI values. This step allowed us to capture the subtle changes in vegetation dynamics over time. In the second step, we used different criteria for each algorithm to identify the key timing of phenological events. For HANTS and POLYFIT, we used extremum values (maximum or minimum) of the change rate to extract the SOS and EOS, while the POS was determined as the date with the maximum value in the fitted daily NDVI curve (table S2). For DLOG, specific parameters were used to identify the SOS, POS, and EOS (table S2). Our analysis using three different algorithms consistently captures the spatiotemporal patterns of phenological events across the Northern Hemisphere, albeit with variations in the specific dates of occurrence (figs. S28 and S29). Notably, some algorithms appear better suited to specific ecosystems. For example, Bolton *et al.* demonstrated that their algorithm performed better in ecosystems with pronounced seasonal changes in leaf growth (e.g., deciduous forests) (*71*). By combining the strengths of each algorithm, the multimethod ensemble approach can mitigate inconsistencies arising from their individual representation of phenological information from NDVI growth curves, thereby potentially reducing uncertainties.

To validate our findings based on the NDVI dataset, we also used the land surface phenology product MCD12Q2 version 6.1, which uses time series data from the two-band enhanced vegetation index (EVI2) and uses a different phenological extraction method compared to ours (https://lpdaac.usgs.gov/products/mcd12q2v061/). Specifically, the green-up in MCD12Q2 version 6.1 is defined as the date when EVI2 first crossed 15% of the segment EVI2 amplitude, the peak is defined as the date when EVI2 reached the segment maximum, the senescence is defined as the date when EVI2 last crossed 90% of the segment EVI2 amplitude, and the dormancy is defined as the date when EVI2 last crossed 15% of the segment EVI2 amplitude. We adopted both the senescence and dormancy to separately represent EOS and verified the results.

In addition, we also used ground-based greenness index [the green chromatic coordinate (GCC)] from PhenoCam Dataset v2.0 and gross primary productivity (GPP) data from FLUXNET2015 Tier 2 as additional verification. The PhenoCam Dataset v2.0 provides a time series of GCC and vegetation phenological observations mainly distributed in North America from 2000 to 2018 (https://daac.ornl.gov/cgi-bin/dsviewer.pl?ds_id=1674) (*72*, *73*). The greenness rising and greenness falling transition dates with 10% of the GCC amplitude of that stage were used as SOS and EOS (*72*, *73*). The POS was the date of maximum GCC extracted from smoothed GCC mean values. The daily GPP data were obtained from the FLUXNET2015 Tier 2 dataset, which consists of flux measurements from various sites worldwide (http://fluxnet.fluxdata.org/data/fluxnet2015-dataset/). We used the following four criteria to select sites for analysis: (i) sites were restricted to the Northern Hemisphere (>30°N); (ii) sites lacking seasonal variation (e.g., evergreen needle-leaf forests and evergreen broadleaf forests) or dominated by human activities (e.g., croplands) were excluded; (iii) only sites with a minimum observation period of 5 years were considered; (iv) sites with phenological dates or ratios exhibiting extreme deviations (>3 SDs) were excluded as outliners (*8*). This selection process resulted in 50 sites from the original 393 sites in the GCC dataset and 49 sites from the original 212 sites of FLUXNET2015 dataset. Details regarding these selected sites can be found in table S1.

We also used monthly GPP outputs from 13 TRENDY v9 S3 simulations (dynamic CO_2_, climate, and land use) (*74*). These 13 models included CABLE-POP, CLASSIC, CLM5.0, IBIS, ISAM, ISBA-CTRIP, JULES-ES-1p0, LPJ-GUESS, LPX-Bern, OCN, ORCHIDEEv3, SDGVM, and VISIT (table S3). These modeled GPP datasets had a time span from 1700 (1900/1860) to 2019 and divergent spatial resolution (table S3); we then used the overlapping time period of 2001 to 2019 and rescaled the spatial resolution to 0.5°. Same methods were used to extract timings of the three phenological events to estimate the ability of these models to capture the observed carryover effects.

To ensure data consistency, we excluded areas with low seasonal dynamics (e.g., barren land) and those subject to intense human interference (e.g., cropland) based on the vegetation classifications provided by the MCD12C1 version 6 product. This product can be accessed at the following link: https://lpdaac.usgs.gov/products/mcd12c1v006/.

Last, to facilitate data integration and analysis, all satellite-based datasets were rescaled to a spatial resolution of 0.5°, aligning them with the environmental data used in our study.

### Environmental datasets

We obtained the monthly temperature, precipitation, and cloud cover data from the Climatic Research Unit gridded time series (CRU TS) version 4.05 dataset (*75*). These data were downloaded from the following source: https://crudata.uea.ac.uk/cru/data/hrg/cru_ts_4.05/. The CRU TS dataset provides spatially gridded information with a resolution of 0.5° and covers the time period from 1901 to 2020.

We used cloud cover as a proxy for solar radiation. We considered cloud cover as an important environmental factor affecting vegetation phenology. The CRU TS dataset provided us with the necessary cloud cover data, which we resampled to an annual mean temporal resolution.

In addition, we obtained the annual mean CO_2_ data for northern ecosystems from the National Oceanic and Atmospheric Administration (NOAA). The data were obtained from the following source: https://gml.noaa.gov/ccgg/mbl/data.php. The NOAA dataset provides information on the annual mean CO_2_ levels and aligns with the time period covered in our phenological analysis.

All the environmental datasets used in our study had the same study period, which matched the duration of the phenological analysis.

### Data analysis

We used general linear and piecewise linear (or nonlinear) regression models to investigate changes in the time allocation pattern over the study period. To examine the variation in the allocation ratio over time, we initially constructed a linear model using the lm() function in R (v4.1.2). This model related the time allocation ratio to the year as a predictor. In addition, we used the “segmented” package in R to perform the piecewise linear regression (*76*). The general linear model was first fitted for each pixel using the lm() function, and then the Davies’ test was used to identify potential breakpoints in the linear model. A breakpoint was considered to exist if the *P* value from the Davies’ test was less than 0.05. Conversely, if the *P* value exceeded 0.05, it indicated that no breakpoint was present in the model. In cases where both models (i.e., general linear and piecewise linear regression models) were statistically significant at the 0.05 level, we compared their performance using the AICc. A smaller AICc value indicated a better-fitting model. Throughout our study, a significance level of *P* < 0.05 was used unless otherwise specified.

To analyze the relationships among timings of the three phenological events more comprehensively, we used PLS-PM to decompose the direct and indirect effects of each factor on phenology. PLS-PM is a causal modeling technique that combines principal components analysis, canonical correlation analysis, and multiple regression. It enables us to summarize multiple observed variables into latent variables, reducing complexity and collinearity among variables. The main procedure involves extracting principal components from a subset of observed variables to form latent variables and optimizing the weights of these components to maximize the predictive power of the model. We implemented PLS-PM using the “plspm” package in R (https://github.com/gastonstat/plspm). Modeled phenology based on GPP dataset from TRENDY v9 models was similarly analyzed as observed dataset.

Several parameters are used to assess the effectiveness of the model. First, loading values represent the coefficients of the linear combinations of observed variables for the latent variables. Higher loading values indicate a greater contribution of the observed variables to the latent variables. Second, *R*^2^ (*R*-squared) is the coefficient for the endogenous variables, indicating how well they are explained by the model. An *R*^2^ value greater than 0.3 is typically considered acceptable. Third, the goodness of fit (GOF) evaluates the overall performance of the model. A higher GOF indicates better model fit.

In addition to PLS-PM, we used partial correlation analysis to assess the direct effect of each factor on phenology while excluding the direct effects of other factors. This analysis allows us to disentangle the specific impact of each factor on phenology.

We investigated if the quantitative relationship among timings of phenological events could enhance our ability to predict phenology under changing climate conditions. To achieve this, we used the ratio of time allocation, combined with spring and summer phenology, to predict VSS in autumn. Predicted EOS can be expressed as: $\text{EOS}=\frac{\left(1+\text{ratio}\right)\times \text{POS}-\text{SOS}}{\text{ratio}}$ . Timing of autumn phenology is influenced by multiple factors and is known to be challenging to predict. Therefore, we used a leave-one-out cross-validation analysis (LOOCV) due to the limited sample size (*n* is equal to or less than 20) for each pixel.

During the LOOCV analysis, we averaged 19 out of 20 ratios to obtain the parameter for the phenological model. The remaining sample’s SOS and POS were then used as input data to fit the model, resulting in the estimation of the autumn phenology. This process was repeated 20 times, iterating from the first to the last sample. Last, we evaluated the performance of the phenological model by examining the linear relationship between the simulated (sim) and observed (obs) autumn phenology as well as calculating the RMSE $\left[\text{RMSE}=\sqrt{\frac{\sum_{i=1}^{n}{\left({\text{obs}}_{i}-{\text{sim}}_{i}\right)}^{2}}{n}}\right]$, *n* is the number of observations and *i* is the *i*th observation.

Furthermore, we assessed the relative importance of vegetation productivity and early timing of phenological events on EOS. Vegetation productivity was represented by the maximum SIF recorded during the growing season for each pixel. We conducted simple correlation analyses to examine the effects of these two factors on EOS and compared the magnitudes of their absolute correlation coefficients (|*r*|). A larger |*r*| value indicates a stronger effect between the two factors.
