# Supplementary material for: Consistent time allocation fraction to vegetation green-up versus senescence across northern ecosystems despite recent climate change
Source: Sci Adv. 2024 Jun 7;10(23):eadn2487. doi: 10.1126/sciadv.adn2487 (PMC11160464; doi:10.1126/sciadv.adn2487)
Supplement: Supplementary file 1 — Figs. S1 to S29 Tables S1 to S3 [file sciadv.adn2487_sm.pdf]

Science Advances
AAAS

Supplementary Materials for

Consistent time allocation fraction to vegetation green-up versus senescence across northern ecosystems despite recent climate change

Fandong Meng *et al.*

Corresponding author: Anping Chen, anping.chen@colostate.edu; Fandong Meng, mengfandong@itpcas.ac.cn

Sci. Adv. **10**, eadn2487 (2024)
DOI: 10.1126/sciadv.adn2487

This PDF file includes:

Figs. S1 to S29
Tables S1 to S3

Supplementary figures

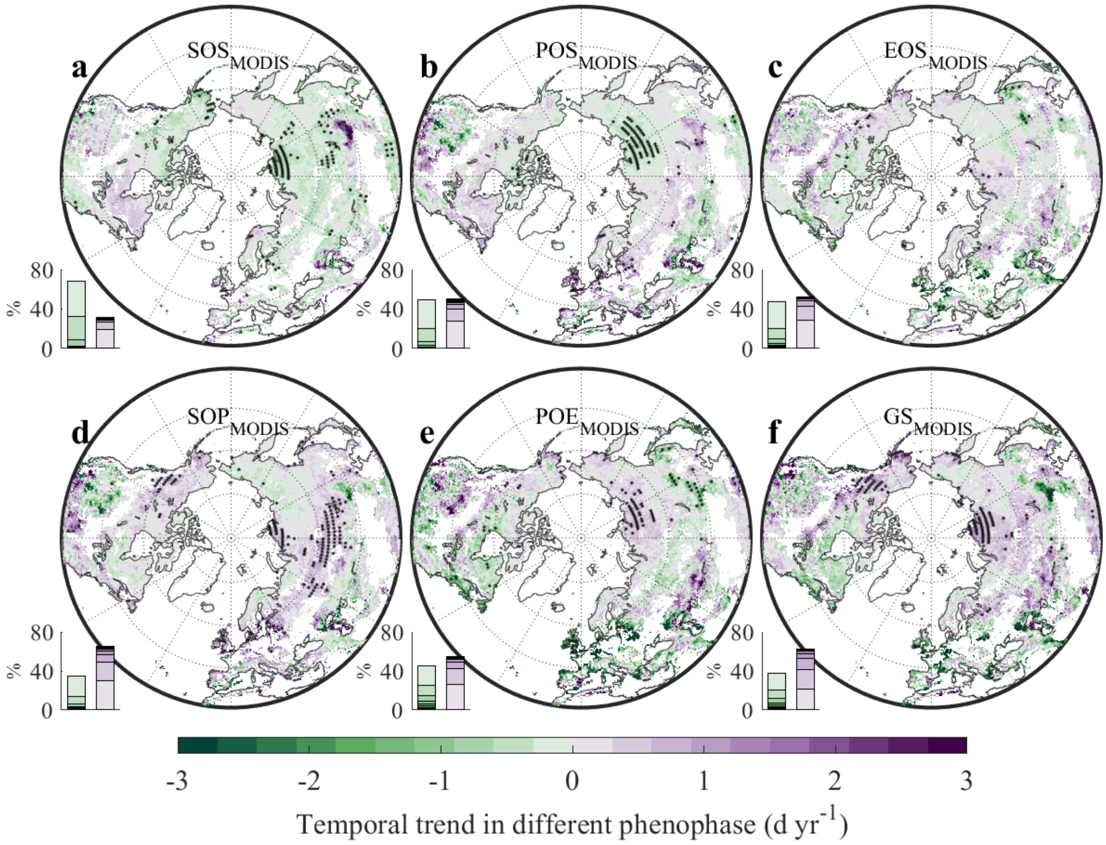

Fig. S1. Temporal trend in different phenological events. The phenological events include start (SOS) (a), peak (POS) (b) and end of growing season (EOS) (c). The vegetation green-up and vegetation senescence were defined as the durations between POS and SOS (SOP) (d) and between EOS and POS (POE) (e), respectively. The GS indicates the total growing season. The inserted histograms are the frequency distribution of temporal trend in growing season from 2001 to 2020 on the Northern Hemisphere.

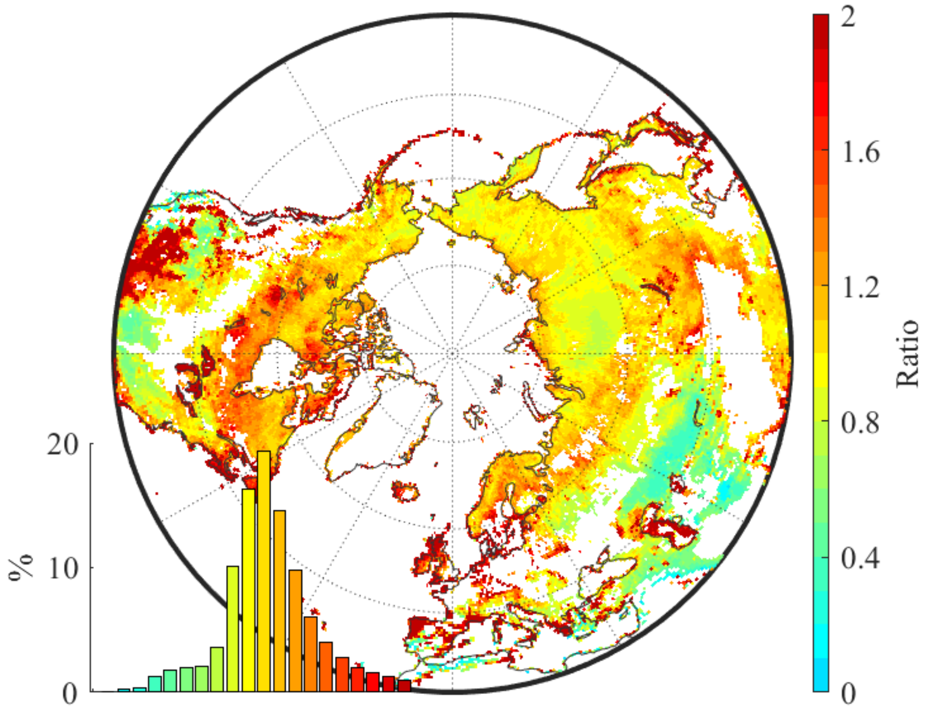

Fig. S2. Spatial pattern of time allocation strategy. The inserted histograms are the frequency distribution of each time allocation strategy. SOS, POS and EOS are the start of growing season, peak of growing season and end of growing season, respectively. Ratio is the time allocation between vegetation green-up and vegetation senescence.

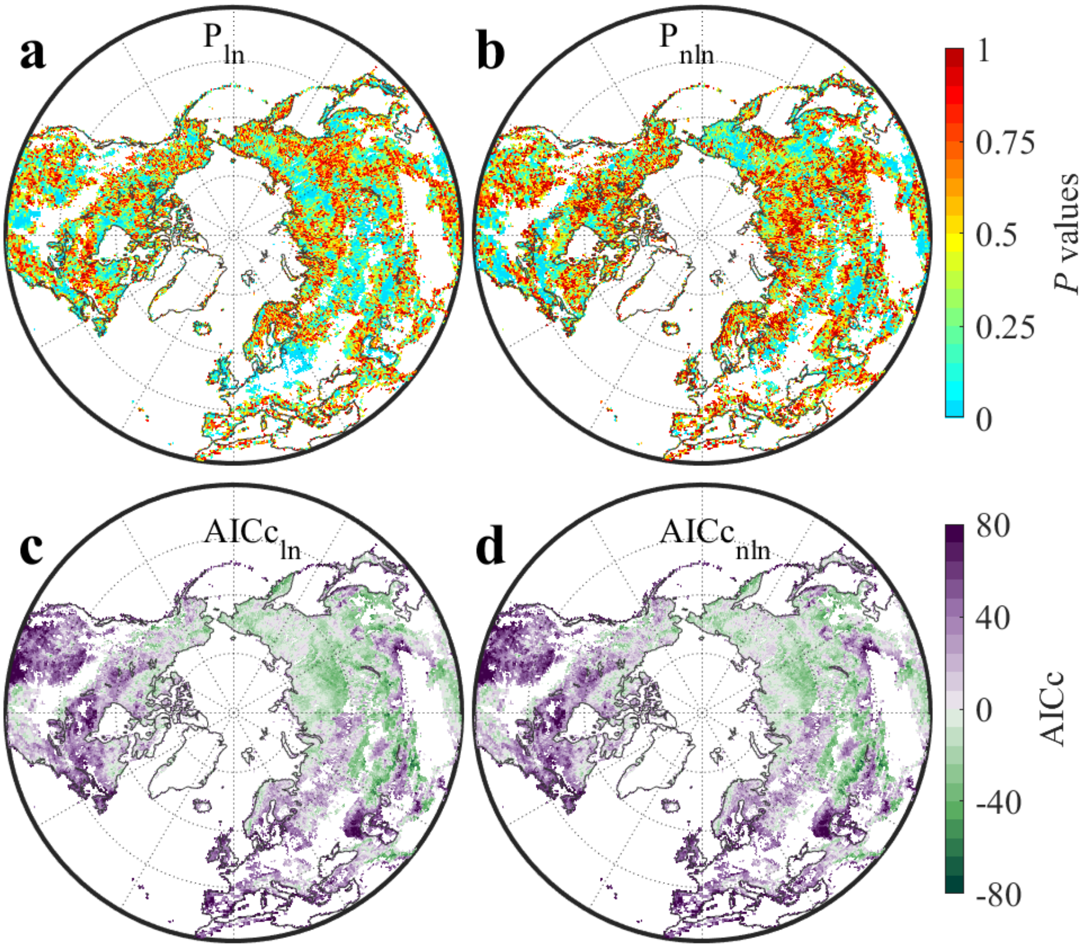

Fig. S3. The *P* values and AICc associated with temporal trend in time allocation strategy in Figure 1. The subscripts of ln and nln represent general linear regression and piecewise linear regression.

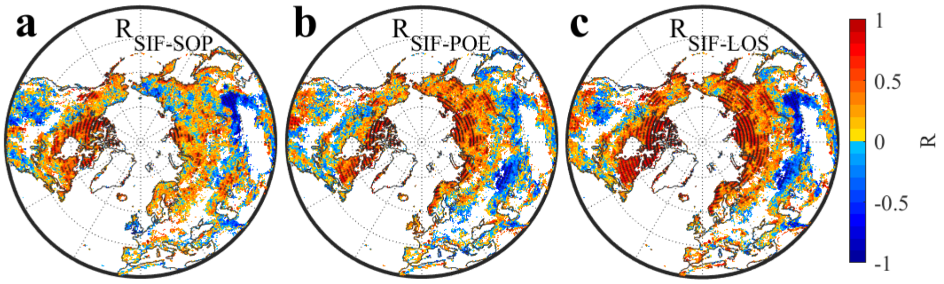

Fig. S4. Spatial pattern of correlation analysis between different phenological metrics and SIF (a proxy of productivity) over the period of 2001–2020. a, correlation analysis between annual mean SIF and SOP with a positive relationship covered 71.4% regions (17.9% at a significant level); b, correlation analysis between annual mean SIF and POE with a positive relationship covered 72.4% regions (24.2% at a significant level); c, correlation analysis between annual mean SIF and LOS with a positive relationship covered 78.3% regions (35.7% at a significant level). Black dots mark significant trends at 0.05 level.

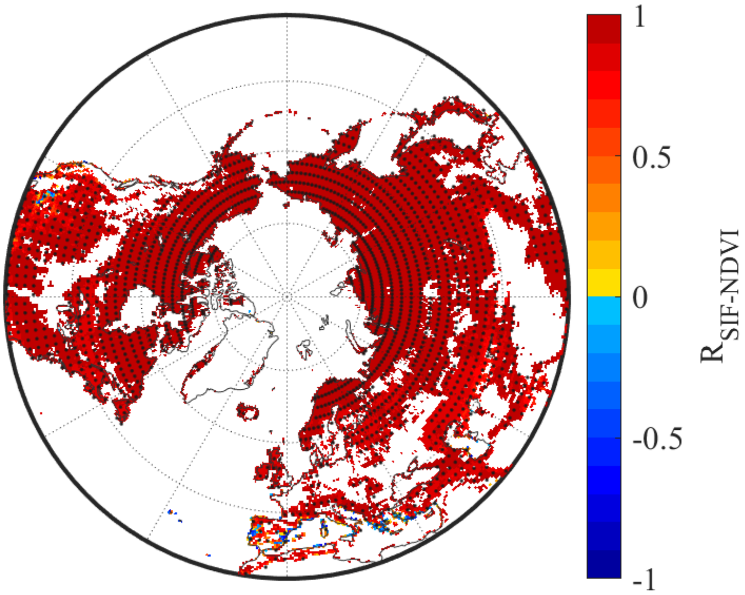

Fig. S5 Spatial pattern of correlation analysis between daily SIF and daily NDVI values over the period of 2001–2020. The SIF and NDVI values are fitted daily curves based on three phenological algorithms and then are averaged. They had a positive relationship covered 99.4% regions (99.3% at a significant level). Black dots mark significant trends at 0.05 level.

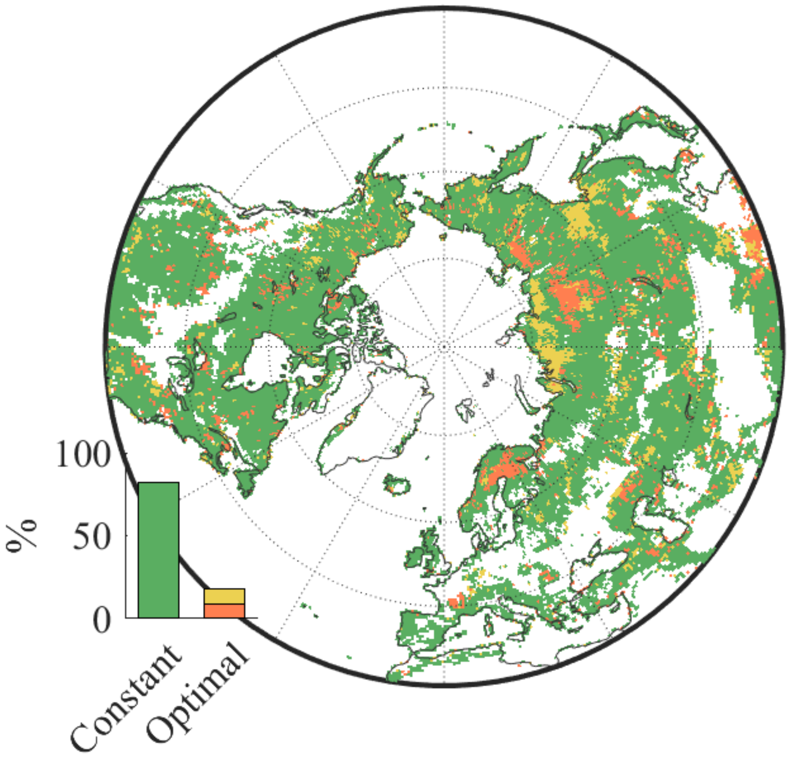

Fig. S6. The different temporal trend of vegetation time allocation for each grid based on SIF. The constant, linear and nonlinear indicate the constant time allocation strategy and the optimal time allocation strategy. The inserted histograms are the frequency distribution of each time allocation strategy. Details related to three time allocation strategies are described in introduction.

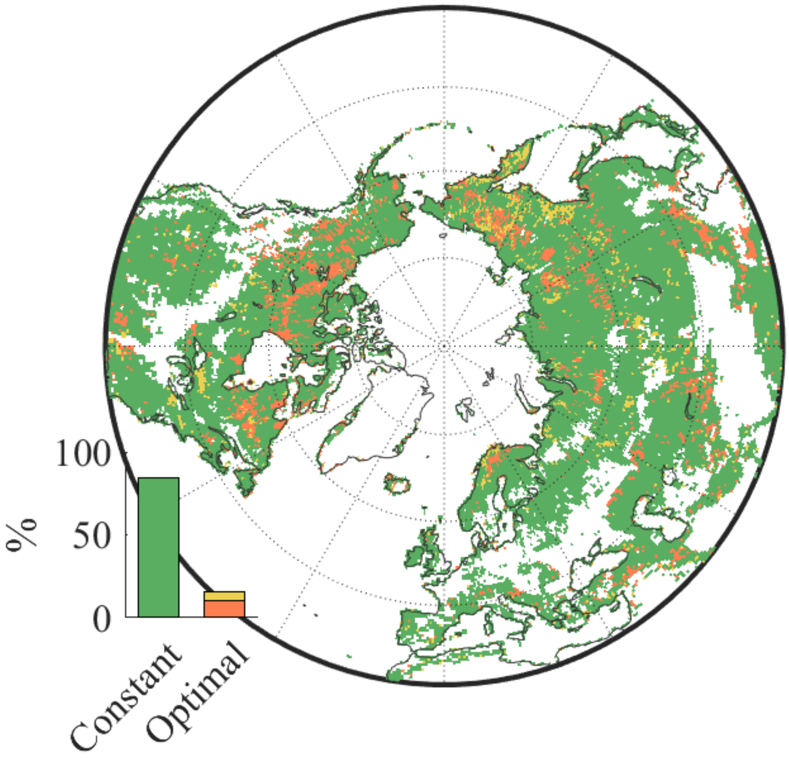

Fig. S7. The different temporal trend of vegetation time allocation for each grid based on GIMMS NDVI. The constant, linear and nonlinear indicate the constant time allocation strategy and the optimal time allocation strategy. The inserted histograms are the frequency distribution of each time allocation strategy. Details related to three time allocation strategies are described in introduction.

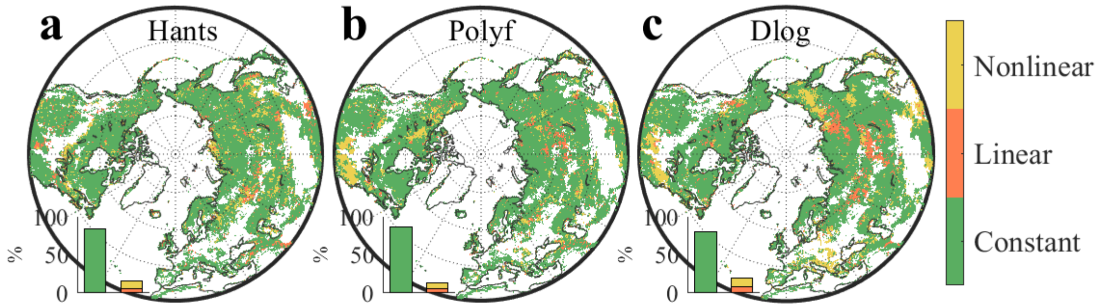

Fig. S8. Temporal trend in time allocation strategy based on different phenological extraction algorithms including Hants (a), Polyf (b) and Dlog (c). Temporal trend in vegetation time allocation between vegetation green-up and vegetation senescence based on NDVI. The inserted histograms are the frequency distribution of each time allocation strategy. Details related to three time allocation strategies are described in introduction.

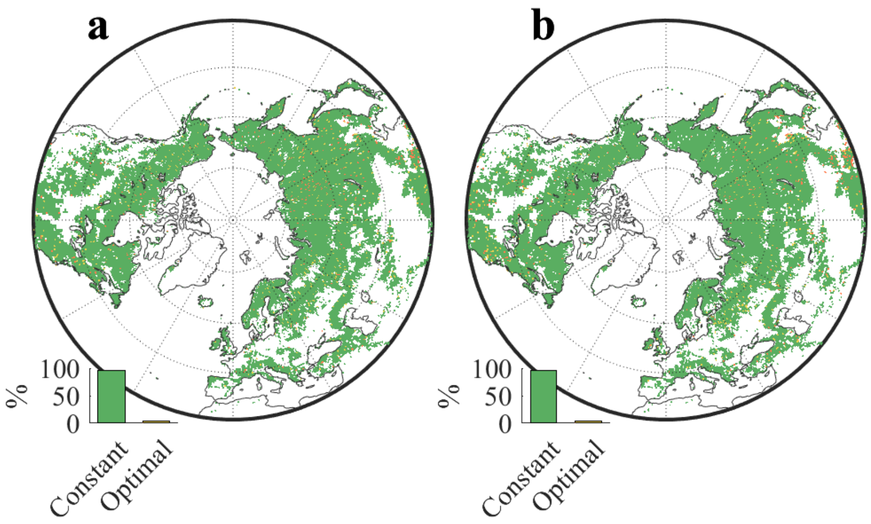

Fig. S9. The different temporal trends of vegetation time allocation for each pixel. The constant and optimal time allocation strategies are represented by blue and red/yellow colors, respectively. Blue indicates non-significant trends, while red/yellow represents significant linear/nonlinear trends. The inserted histograms display the frequency distribution of each time allocation pattern. a, the time allocation is calculated by the ratio between (peak - greenup)/(senescence-peak); b, the time allocation is calculated by the ratio between (peak - greenup)/(dormancy-peak).

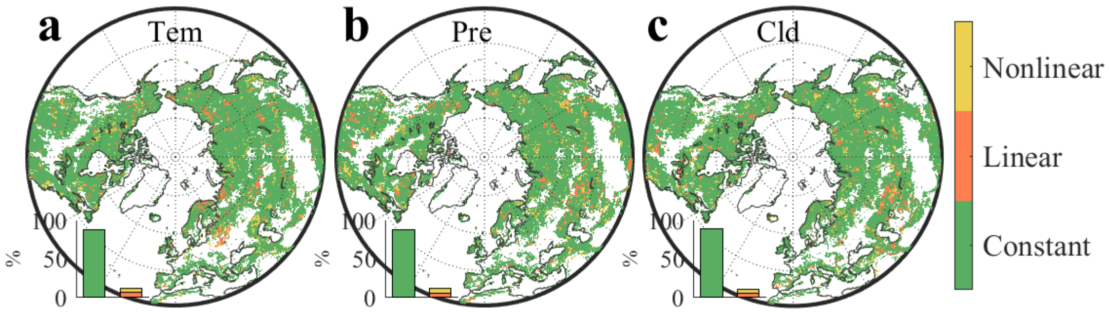

Fig. S10. Trend in time allocation strategy based on different environmental factors including temperature (Tem, a), precipitation (Pre, b) and cloud cover (Cld, c). The inserted histograms are the frequency distribution of each time allocation strategy. Details related to three time allocation strategies are described in introduction.

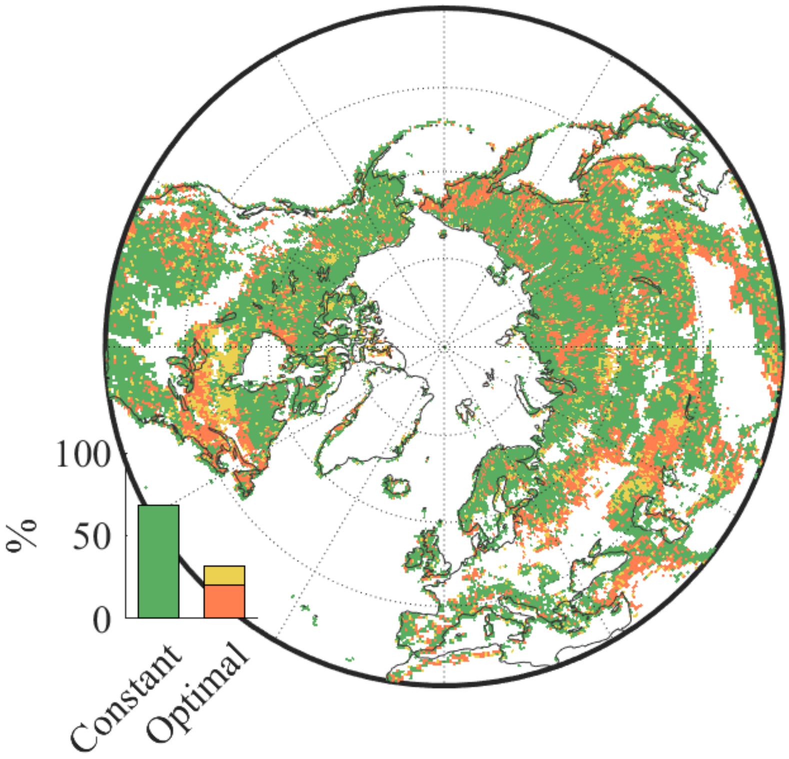

Fig. S11. Trend in time allocation strategy based on growing season length. The inserted histograms are the frequency distribution of each time allocation strategy.

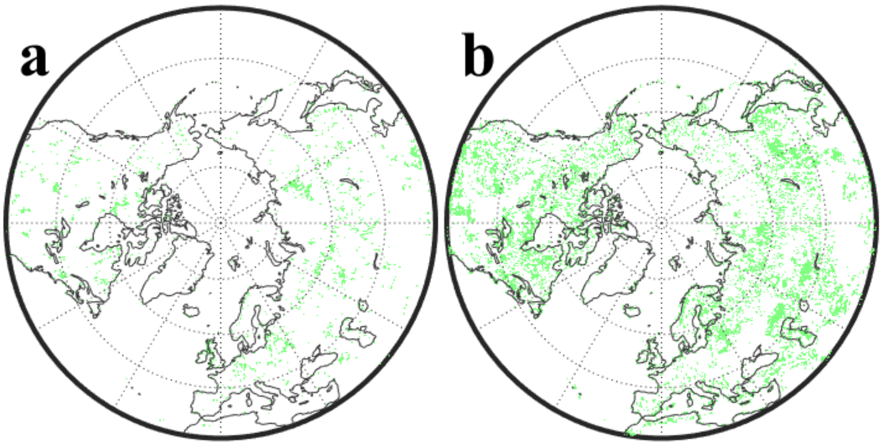

Fig. S12. The stationarity test of time series between two periods. The comparison of mean values (a) and standard deviations (b) between two periods (2001-2010 vs. 2011-2020). The blue colors indicate significant difference at 0.05 level for each pixel, the rest regions are shown in white colors.

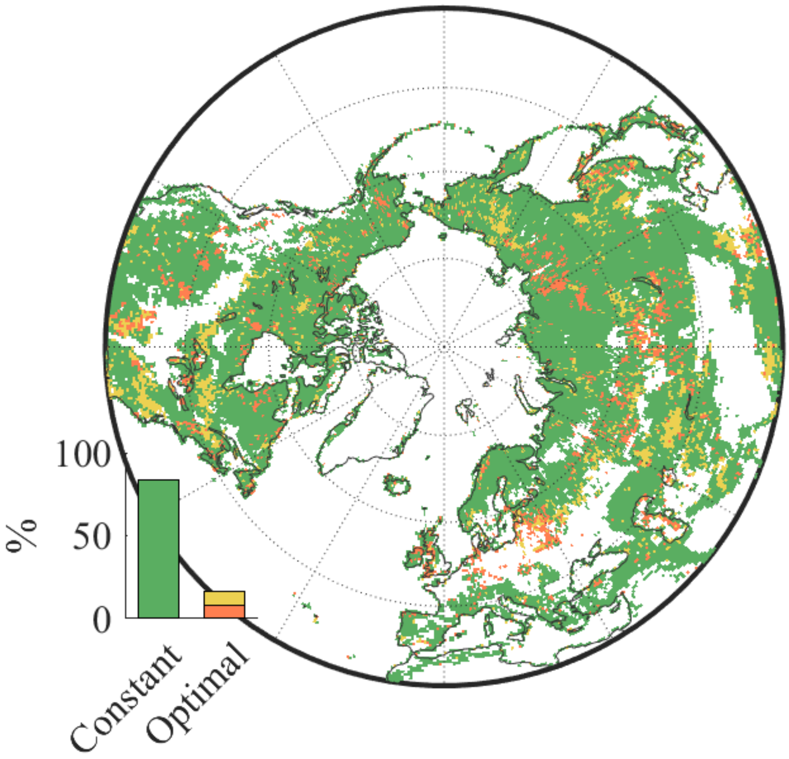

Fig. S13. The different temporal trend of vegetation time allocation for each grid based on anomaly. The constant, linear and nonlinear indicate the constant time allocation strategy and the optimal time allocation strategy. The inserted histograms are the frequency distribution of each time allocation strategy. Details related to three time allocation strategies are described in introduction.

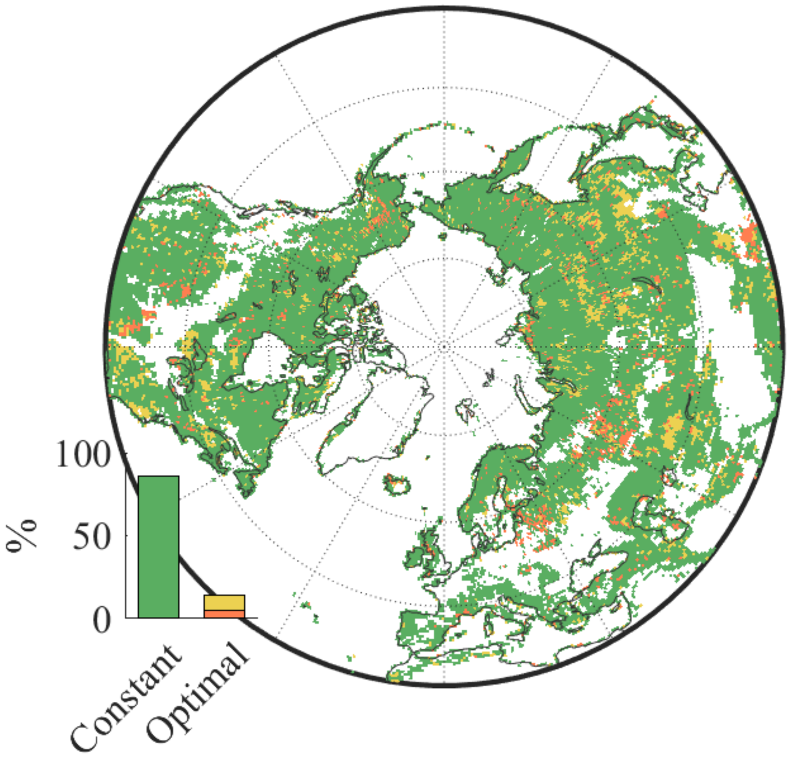

Fig. S14. The different temporal trend of vegetation time allocation for each grid by excluding the steady period in leaf life span. The constant, linear and nonlinear indicate the constant time allocation strategy and the optimal time allocation strategy. The inserted histograms are the frequency distribution of each time allocation strategy. Details related to three time allocation strategies are described in introduction.

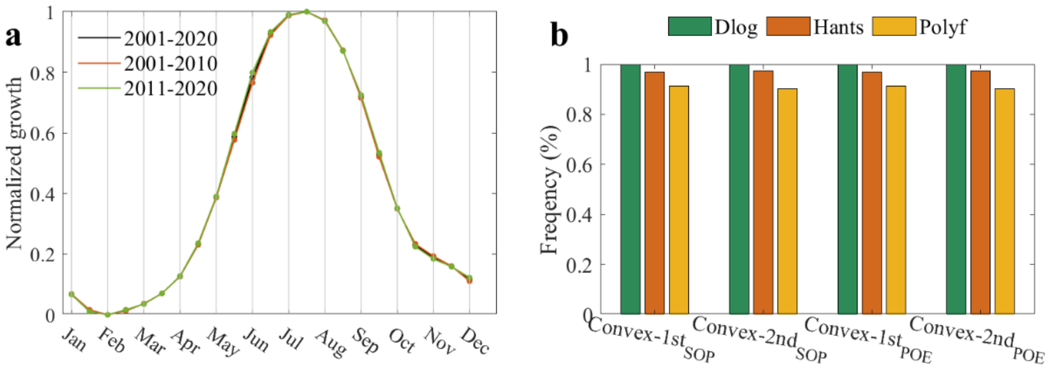

Fig. S15. Characteristics of the shape of NDVI curve and its changes over years. a, changes in normalized NDVI over the Northern Hemisphere. The normalized NDVI curve is derived from the multi-year average for the period 2000–2020 (black line), 2000–2010 (red line) and 2011–2020 (green line); b, the shape of NDVI curve is estimated by the sign of its second derivative in the fitted daily NDVI curve based on three algorithms. The negative and positive signs represent convex and concave curve, respectively. The vegetation green-up and vegetation senescence were defined as the durations between POS and SOS (SOP) (d) and between EOS and POS (POE) (e), respectively. The convex-1st and convex-2nd represent the shape of NDVI curve during two periods (2000–2010 vs 2011–2020).

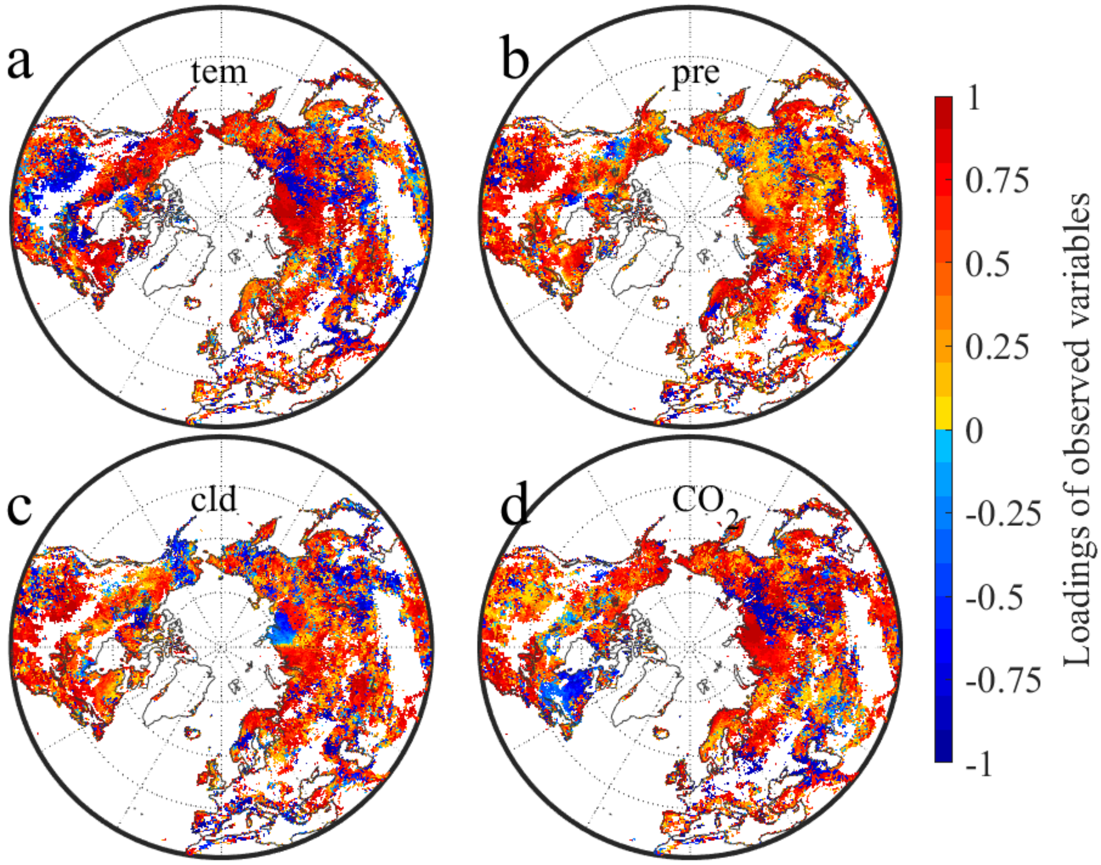

Fig. S16. The loadings of climatic factors in the PLS-PM analysis. tem, pre, cld and CO_2 indicate annual mean temperature, precipitation, radiation and CO_2. The loading values are the correlation between the latent variable and its observation variables. The higher the correlation, the better the construction of the latent variable.

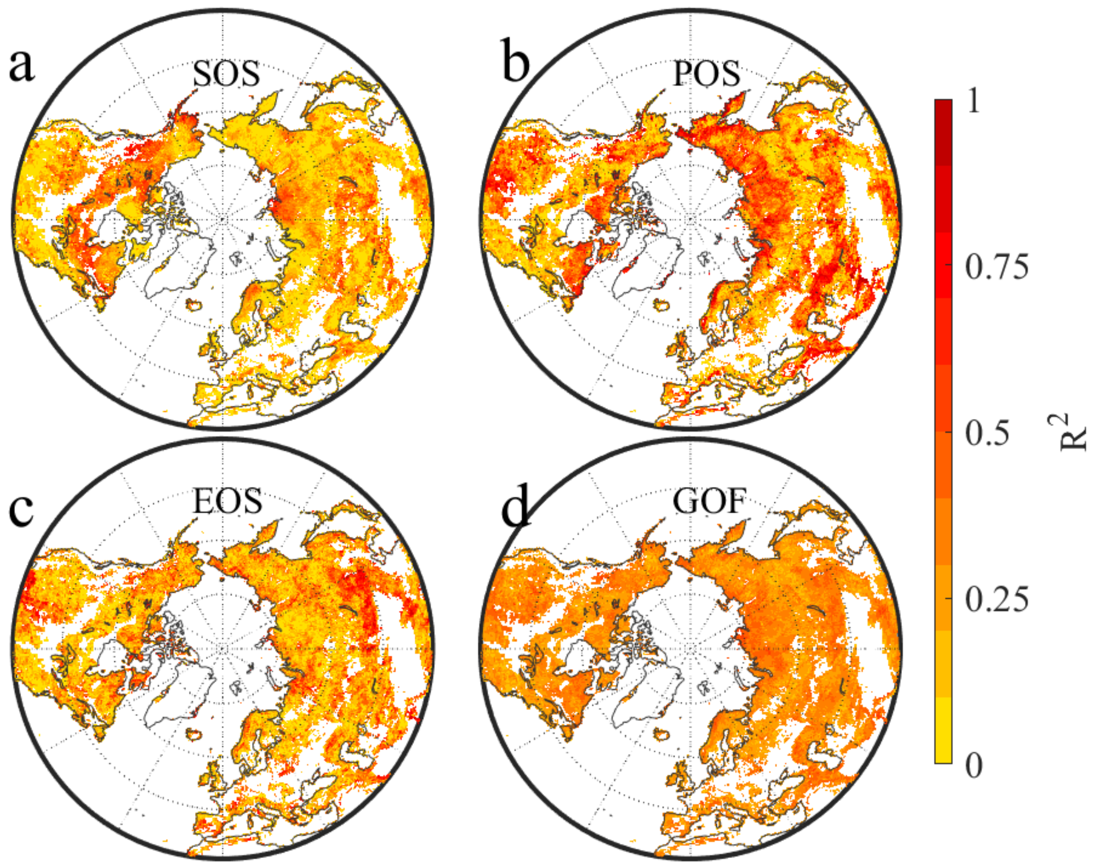

Fig. S17. The coefficient for endogenous variables and goodness of fit (GOF) in the PLS-PM analysis. SOS is start of growing season, POS is peak of growing season, and EOS is end of growing season.

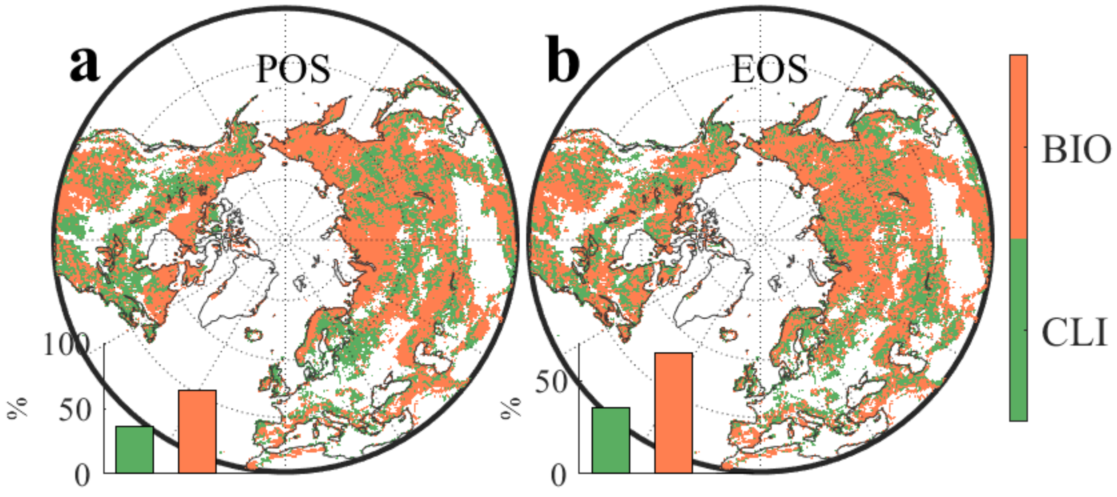

Fig. S18. The dominant factors of POS and EOS based on PLS-PM analysis. CLI includes annual mean temperature, precipitation, radiation and CO_2. BIO indicates SOS for POS, and SOS and POS for EOS. SOS is start of growing season, POS is peak of growing season, and EOS is end of growing season. The relative importance of each factor is the absolute value of path correlations.

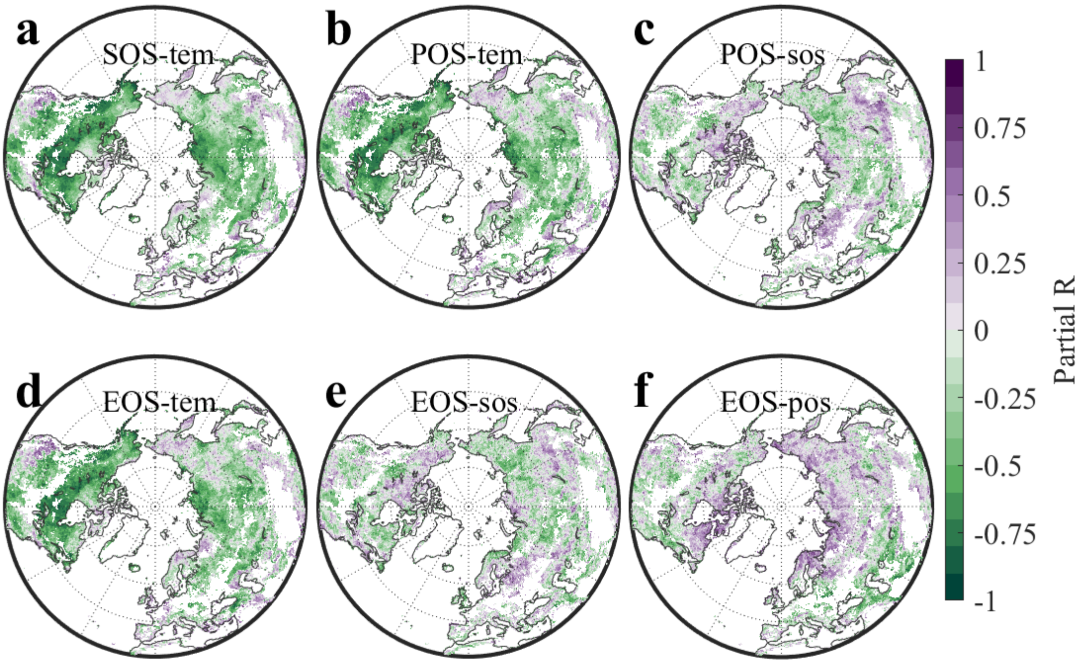

Fig. S19. The partial correlation coefficient of each factor on three phenological events by partial correlation analysis. The capitalized words are the dependent factors and the non-capitalized words are the independent factors. Tem is annual mean temperature, SOS is start of growing season, POS is peak of growing season, and EOS is end of growing season. Here we only used annual mean temperature to exclude overfitting in partial correlation analysis.

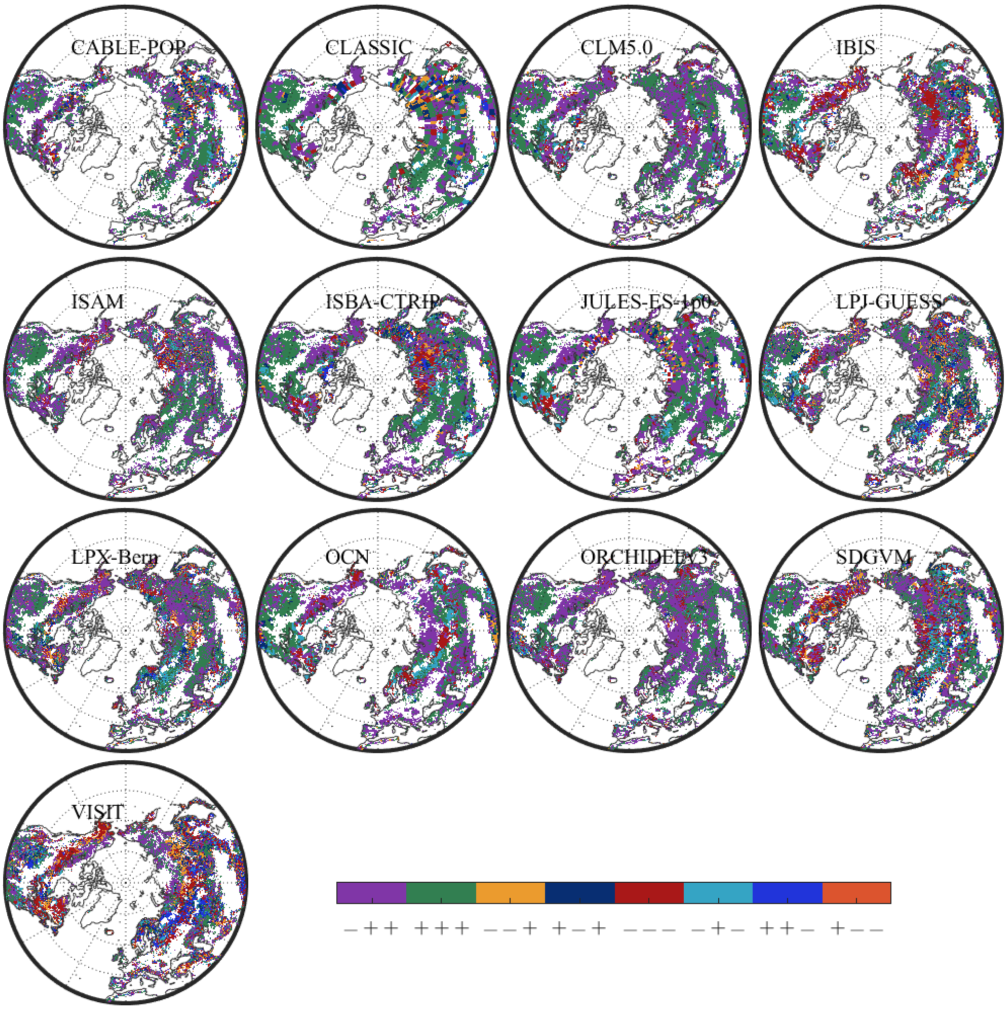

Fig. S20. Spatial pattern of carryover effect of CLI→SOS→POS→EOS for 13 trendy models. For example, the '− + +' represents negative correlations between climate variables and SOS (CLI→SOS), positive correlations between SOS and POS (SOS→POS) and POS and EOS (POS→EOS).

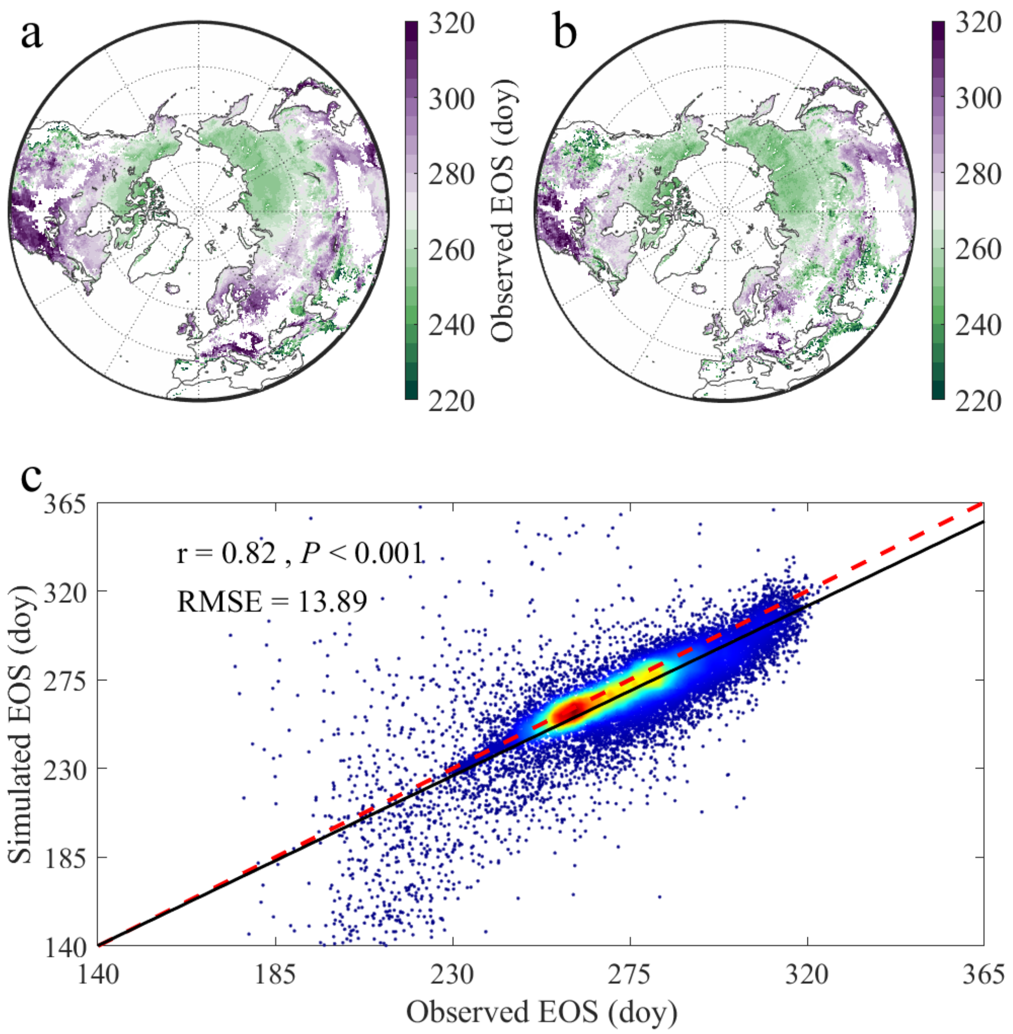

Fig. S21. Spatial pattern of EOS on the Northern Hemisphere. Spatial pattern of observed EOS (a) and simulated EOS (b). (c), The simple linear relationship between observed and simulated EOS on the Northern Hemisphere. The color gradients from cold to warm represent increased density of points. RMSE (Root Mean Squared Error) is used to estimate the performance of model. The significant level is at 0.05 level. The significant level is at 0.05 level.

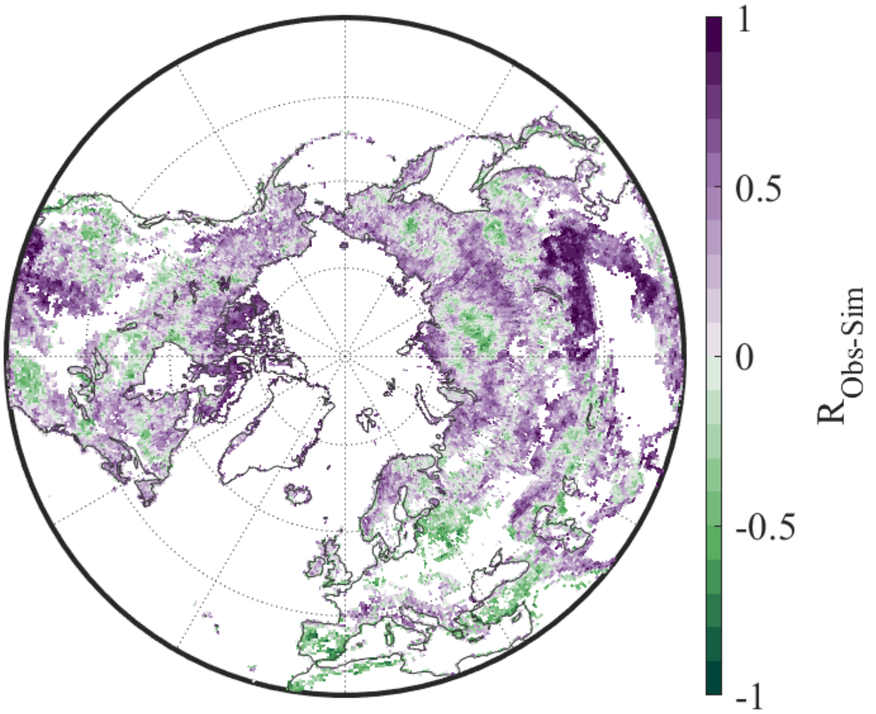

Fig. S22. Spatial pattern of correlation coefficient between observed and simulated EOS. The positive values indicate a similar temporal trend between observed and simulated EOS, the negative values indicate an opposite temporal trend between observed and simulated EOS.

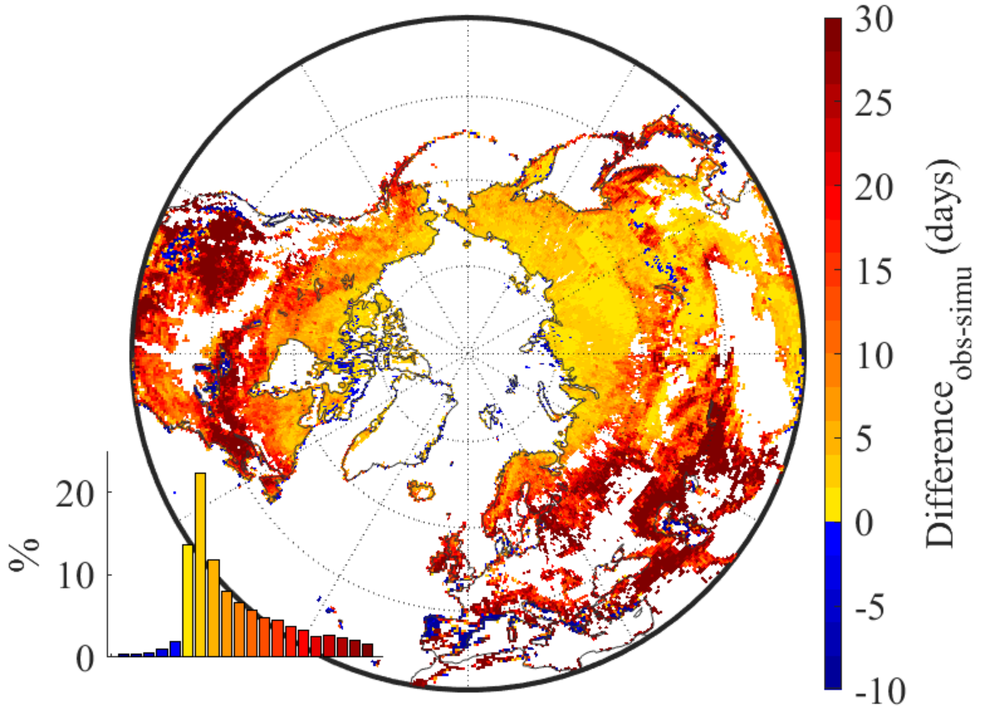

Fig. S23. Spatial pattern of difference between observed and simulated EOS. The positive values indicate the underestimated EOS by model, otherwise, the simulated EOS is overestimated. The inserted histograms are the frequency distribution of each bin of difference values.

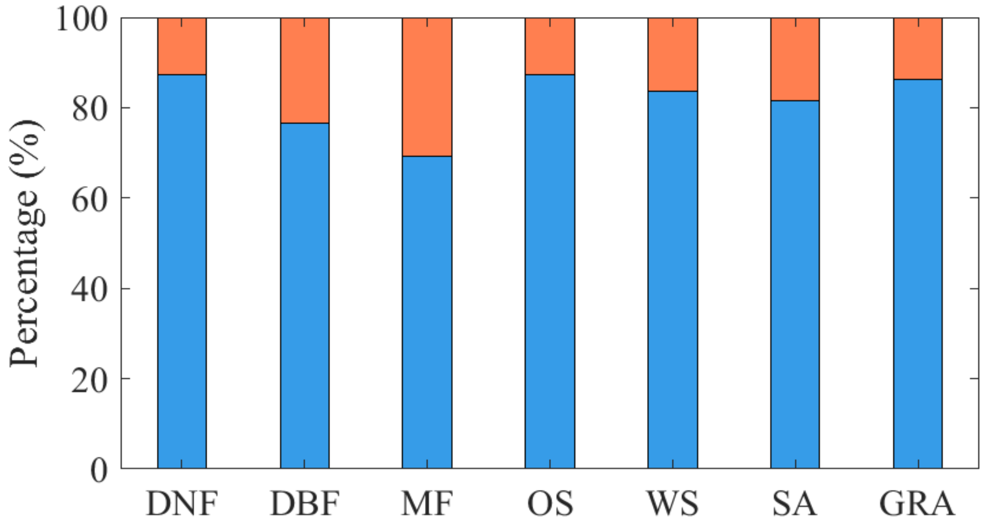

FIG. S24. The percentage of two time allocation strategy for different plant functional types. The blue and red colors indicate the percentage of the constant and optimal time allocation strategies, respectively. DNF: Deciduous Needleleaf Forests; DBF: Deciduous Broadleaf Forests; MF: Mixed Forests; OS: Open Shrublands; WS: Woody Savannas; SA: Savannas; GRA: Grasslands.

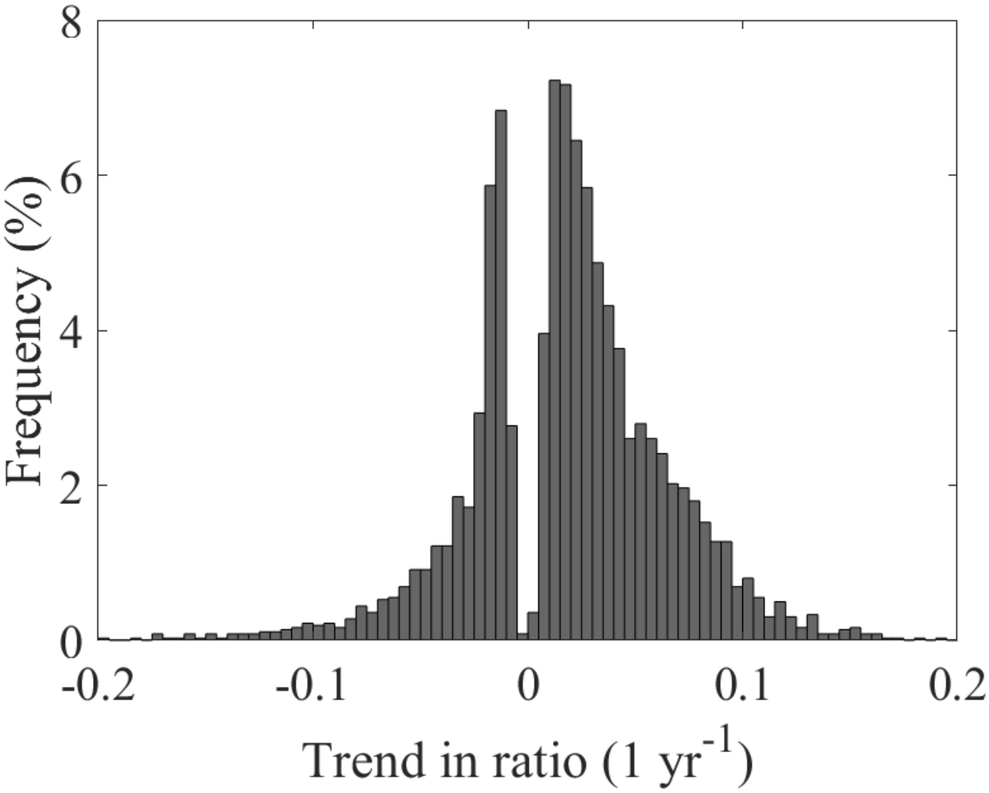

FIG. S25. The frequency of temporal trends in the optimal time allocation strategy. We used the linear slopes here because it was more simpler with only one trend compared with nonlinear slopes. The percentages of optimal time allocation were 31.3% with negative values indicating more time allocated to vegetation senescence and 68.7% with positive values indicating more time allocated to vegetation green-up.

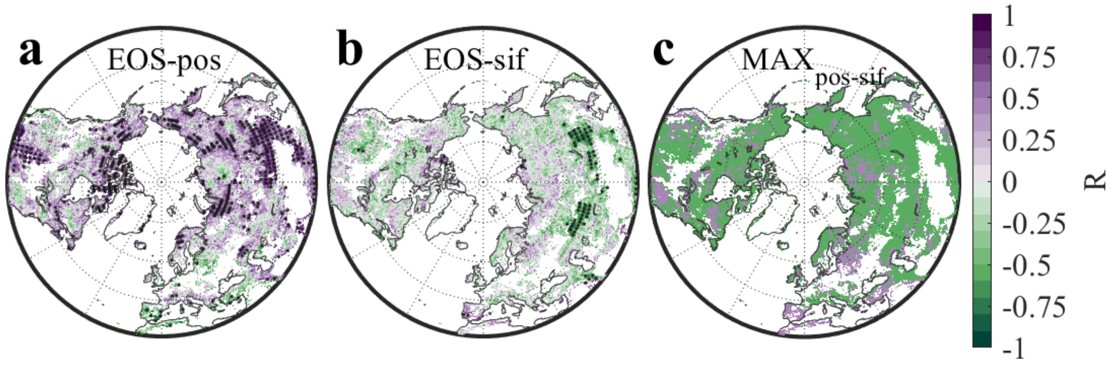

FIG. S26. The comparison between effects of POS and SIF on EOS. (a), The correlation coefficient between POS and EOS. (b), The correlation coefficient between SIF_{max} and EOS. (c), The relative importance between POS and SIF_{max}. The green and brown colors indicate the effect of POS and SIF_{max}, respectively. The blank dots indicate the significant level at 0.05.

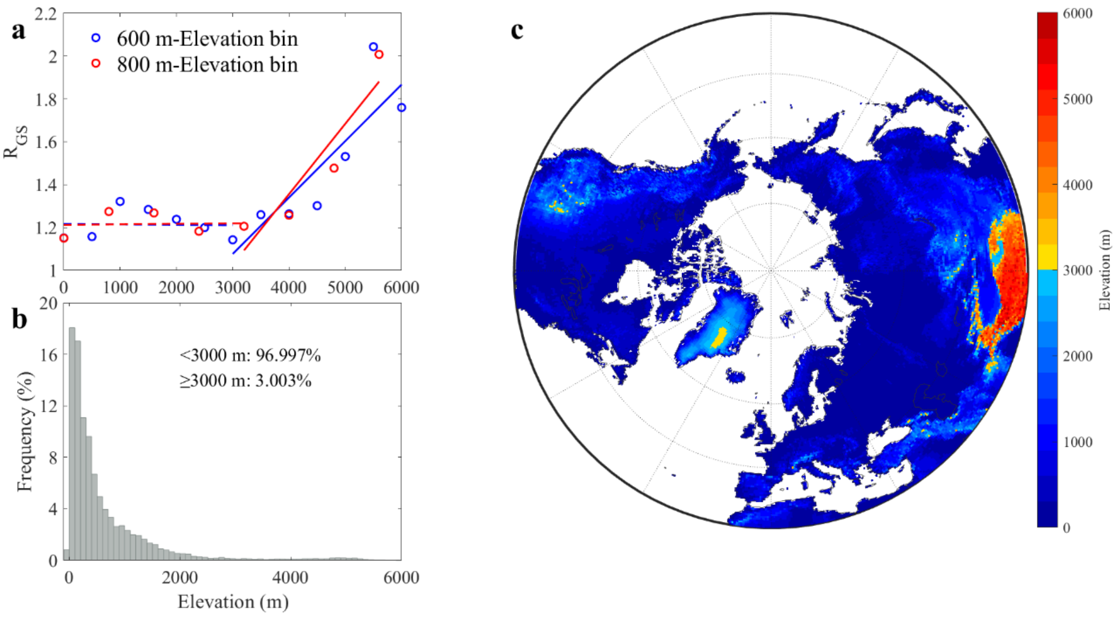

FIG. S27. The relationship between time allocation ratios and elevations across the Northern Hemisphere. a, the nonlinear relationship between elevations and time allocation ratios across the Northern Hemisphere; b, the frequency distribution of elevations across the Northern Hemisphere; c, the spatial pattern of elevations on the Northern Hemisphere. The Digital Elevation Model (DEM) data was acquired from the Shuttle Radar Topographic Mission (SRTM, https://www.earthdata.nasa.gov/sensors/srtm).

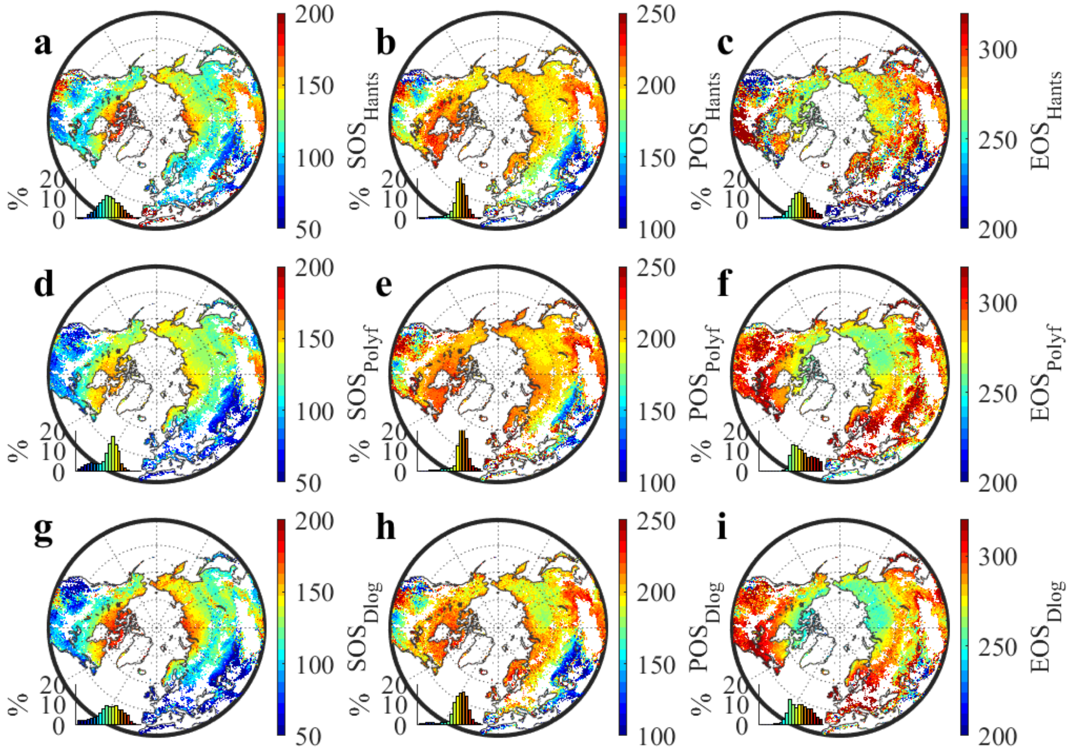

FIG. S28. Spatial patterns of the multi-year mean phenology over the period of 2001–2020. SOS, POS, and EOS indicate the date of start, peak and end of the growing season; and subscripts Hants, Polyf and Dlog are the three phenological extraction algorithms, respectively. The inserted figure in each panel showed its frequency distribution of phenological dates (day of year).

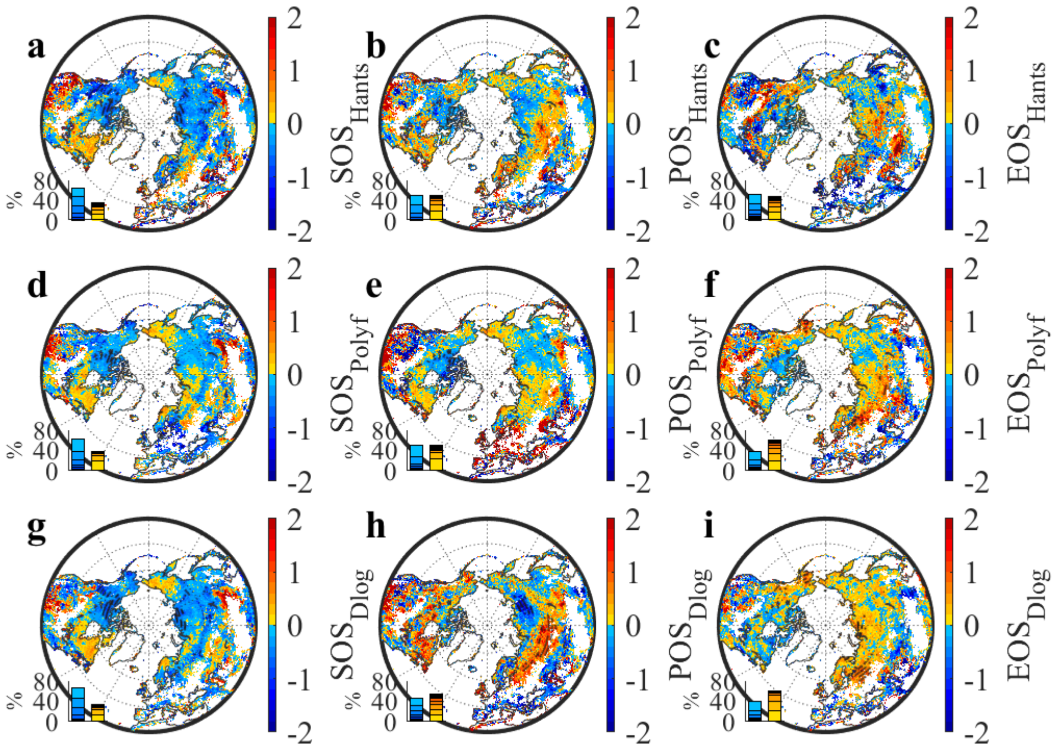

FIG. S29. Temporal trend of phenology over the period of 2001–2020. SOS, POS, and EOS indicate the start, peak and end of the growing season; and subscripts Hants, Polyf and Dlog are the three phenological extraction algorithms, respectively. The inserted figure in each panel showed the frequency distribution of its temporal trends (days per year). Black dots mark significant trends at 0.05 level.

Supplementary table

Table S1. The chosen sites' information of FLUXNET2015 (49) and PhenoCam (50) in this study.

Category	SITE_ID	SITE_NAME	IGBP	LAT	LON
FLUX	AT-Neu	Neustift	GRA	47.12	11.32
FLUX	BE-Bra	Brasschaat	MF	51.31	4.52
FLUX	BE-Vie	Vielsalm	MF	50.30	6.00
FLUX	CA-Gro	Ontario - Groundhog River, Boreal Mixedwood Forest	MF	48.22	-82.16
FLUX	CA-Oas	Saskatchewan - Western Boreal, Mature Aspen	DBF	53.63	-106.20
FLUX	CH-Cha	Chamau	GRA	47.21	8.41
FLUX	CH-Fru	Früebüel	GRA	47.12	8.54
FLUX	CH-Lae	Laegern	MF	47.48	8.36
FLUX	CH-Oe1	Oensingen grassland	GRA	47.29	7.73
FLUX	CZ-BK2	Bily Kriz grassland	GRA	49.49	18.54
FLUX	CZ-wet	Trebon (CZECHWET)	WET	49.02	14.77
FLUX	DE-Akm	Anklam	WET	53.87	13.68
FLUX	DE-Gri	Grillenburg	GRA	50.95	13.51
FLUX	DE-Hai	Hainich	DBF	51.08	10.45
FLUX	DE-Lnf	Leinefelde	DBF	51.33	10.37
FLUX	DE-Spw	Spreewald	WET	51.89	14.03
FLUX	DK-Sor	Soroe	DBF	55.49	11.64
FLUX	ES-LJu	Llano de los Juanes	OSH	36.93	-2.75
FLUX	FR-Fon	Fontainebleau-Barbeau	DBF	48.48	2.78
FLUX	GL-NuF	Nuuk Fen	WET	64.13	-51.39
FLUX	GL-ZaH	Zackenberg Heath	GRA	74.47	-20.55
FLUX	IT-Col	Collelongo	DBF	41.85	13.59
FLUX	IT-MBo	Monte Bondone	GRA	46.01	11.05
FLUX	IT-Noe	Arca di Noe - Le Prigionette	CSH	40.61	8.15
FLUX	IT-Ro1	Roccarespampani 1	DBF	42.41	11.93
FLUX	IT-Ro2	Roccarespampani 2	DBF	42.39	11.92
FLUX	IT-Tor	Torgnon	GRA	45.84	7.58
FLUX	NL-Hor	Horstermeer	GRA	52.24	5.07
FLUX	RU-Cok	Chokurdakh	OSH	70.83	147.49
FLUX	RU-Sam	Samoylov	GRA	72.37	126.50
FLUX	US-Atq	Atqasuk	WET	70.47	-157.41
FLUX	US-Goo	Goodwin Creek	GRA	34.25	-89.87
FLUX	US-Ha1	Harvard Forest EMS Tower (HFR1)	DBF	42.54	-72.17
FLUX	US-IB2	Fermi National Accelerator Laboratory- Batavia (Prairie site)	GRA	41.84	-88.24
FLUX	US-Los	Lost Creek	WET	46.08	-89.98

FLUX	US-MMS	Morgan Monroe State Forest	DBF	39.32	-86.41
FLUX	US-Oho	Oak Openings	DBF	41.55	-83.84
FLUX	US-PFa	Park Falls/WLEF	MF	45.95	-90.27
FLUX	US-SRC	Santa Rita Creosote	OSH	31.91	-110.84
FLUX	US-SRG	Santa Rita Grassland	GRA	31.79	-110.83
FLUX	US-SRM	Santa Rita Mesquite	WSA	31.82	-110.87
FLUX	US-Syv	Sylvania Wilderness Area	MF	46.24	-89.35
FLUX	US-Ton	Tonzi Ranch	WSA	38.43	-120.97
FLUX	US-UMB	Univ. of Mich. Biological Station	DBF	45.56	-84.71
FLUX	US-UMd	UMBS Disturbance	DBF	45.56	-84.70
FLUX	US-Var	Vaira Ranch- Ione	GRA	38.41	-120.95
FLUX	US-WCr	Willow Creek	DBF	45.81	-90.08
FLUX	US-Whs	Walnut Gulch Lucky Hills Shrub	OSH	31.74	-110.05
FLUX	US-Wkg	Walnut Gulch Kendall Grasslands	GRA	31.74	-109.94
PhenoCam	71	Acadia	DBF	-68.26	44.38
PhenoCam	76	Alligatorriver	DBF	-75.90	35.79
PhenoCam	112	Bartlett	DBF	-71.29	44.06
PhenoCam	113	Bartlettir	DBF	-71.29	44.06
PhenoCam	123	Bitterootvalley	DBF	-114.09	46.51
PhenoCam	140	Burnssagebrush	SH	-119.69	43.47
PhenoCam	141	Butte	GRA	-112.48	45.95
PhenoCam	147	Canadaobs	DNF	-105.12	53.99
PhenoCam	148	Canadaobs	ENF	-105.12	53.99
PhenoCam	150	Caryinstitute	DBF	-73.73	41.78
PhenoCam	165	Dollysods	DBF	-79.43	39.10
PhenoCam	169	Downerwoods	DBF	-87.88	43.08
PhenoCam	170	Drippingsprings	DBF	-116.80	33.30
PhenoCam	186	Gatesofthemountains	GRA	-111.71	46.83
PhenoCam	187	Gatesofthemountains	GRA	-111.71	46.83
PhenoCam	202	Harvard	DBF	-72.17	42.54
PhenoCam	203	Harvardbarn2	DBF	-72.19	42.54
PhenoCam	205	Harvardbarn	DBF	-72.19	42.54
PhenoCam	212	Harvardhemlock	DBF	-72.18	42.54
PhenoCam	224	Howland1	ENF	-68.74	45.20
PhenoCam	243	Imcrkridge0	TN	-149.30	68.61
PhenoCam	263	Kansas	GRA	-95.19	39.06
PhenoCam	276	Konza	GRA	-96.56	39.08
PhenoCam	291	Mammothcave	DBF	-86.10	37.19
PhenoCam	307	Missouriozarks	DBF	-92.20	38.74
PhenoCam	315	Monture	DBF	-113.13	47.02
PhenoCam	316	Monture	DBF	-113.13	47.02
PhenoCam	317	Monture	GRA	-113.13	47.02
PhenoCam	318	Monture	GRA	-113.13	47.02

PhenoCam	321	Morganmonroe	DBF	-86.41	39.32
PhenoCam	325	Nationalcapital	DBF	-77.07	38.89
PhenoCam	343	Northattleboroma	DBF	-71.31	41.98
PhenoCam	347	Oakridge1	DBF	-84.33	35.93
PhenoCam	348	Oakridge2	DBF	-84.33	35.93
PhenoCam	350	Oregonmp	ENF	-121.56	44.45
PhenoCam	355	Pointreyes	SH	-123.02	38.00
PhenoCam	357	Proctor	DBF	-72.87	44.53
PhenoCam	361	Readingma	DBF	-71.13	42.53
PhenoCam	388	Shiningrock	DBF	-82.77	35.39
PhenoCam	392	Smokylook	DBF	-83.94	35.63
PhenoCam	396	Smokypurchase	DBF	-83.07	35.59
PhenoCam	398	Smokypurchase	GRA	-83.07	35.59
PhenoCam	401	Snakerivermn	DBF	-93.24	46.12
PhenoCam	460	Teddy	GRA	-103.38	46.89
PhenoCam	467	Tonzi	DBF	-120.97	38.43
PhenoCam	469	Torgnon-Ld	DNF	7.56	45.82
PhenoCam	489	Uiefprairie	GRA	-88.20	40.06
PhenoCam	511	Uwmfieldsta	DBF	-88.02	43.39
PhenoCam	522	Willowcreek	DBF	-90.08	45.81
PhenoCam	526	Woodshole	DBF	-70.64	41.55

Note: GRA, MF, DBF, DNF, ENF, WET, OSH, CSH, SH, TN and WSA signify grasslands, mixed forests, deciduous broadleaf forests, deciduous needleleaf forests, evergreen needleleaf forests, wetlands, open shrublands, closed shrublands, shrubs, tundra and woody savannas, respectively.

Table S2. The algorithms used to extract the three phenological events.

Phenology algorithms	Curve fitted functions	SOS	POS	EOS
HANTS	$NDVI_t = \alpha_0 + \sum_{i=1}^{n} \alpha_i \cos(\omega_i t - \varphi_i)$	Date with the maximum value of first derivative	Date with the peak value	Date with the minimum value of first derivative
Polyfit	$NDVI_t = \alpha_0 + \alpha_1 t + \alpha_2 t^2 + \alpha_3 t^3 + \ldots\ldots + \alpha_n t^6$	Date with the maximum value of first derivative	Date with the peak value	Date with the minimum value of first derivative
Double logistic	$NDVI_t = NDVI_{min} + (NDVI_{max} - NDVI_{min}) * (\frac{1}{1+e^{a(b-t)}} + \frac{1}{1+e^{c(d-t)}} - 1)$	b	Date with the peak value	d

Note: t is the day of year, a_n, b, c and d are the fitted coefficients.

Table S3. Details of the 13 TRENDY V9 models used in this study.

Model	Spatial resolution	Temporal resolution	Time period
CABLE-POP	1°×1°	monthly	1700-2019
CLASSIC	0.3556°×0.3556°	monthly	1701-2019
CLM5.0	1.0667°×0.8°	monthly	1700-2019
IBIS	2°×2°	monthly	1700-2019
ISAM	0.5°×0.5°	monthly	1700-2019
ISBA-CTRIP	1°×1°	monthly	1700-2019
JULES-ES-1p0	0.7889°×0.5333°	monthly	1700-2019
LPJ-GUESS	0.5°×0.5°	monthly	1700-2019
LPX-Bern	1°×1°	monthly	1700-2019
OCN	3.75°×3.75°	monthly	1700-2019
ORCHIDEEv3	0.5°×0.5°	monthly	1700-2019
SDGVM	1°×1°	monthly	1900-2019
VISIT	0.5°×0.5°	monthly	1860-2019
